# Programmable Antigen‐Specific Immunity via Self‐Adjuvanting Nanovaccines Co‐Delivering Immune Modulators

**DOI:** 10.1002/anie.202520474

**Published:** 2025-12-17

**Authors:** Keita Ito, Yoshiyuki Manabe, Shino Ohshima, Masatoshi Maeki, Manabu Tokeshi, Hiroshi Inaba, Kazunori Matsuura, Kazuya Kabayama, Yoshie Kametani, Koichi Fukase

**Affiliations:** ^1^ Department of Chemistry Graduate School of Science The University of Osaka 1‐1 Machikaneyama Toyonaka Osaka 560‐0043 Japan; ^2^ Forefront Research Center The University of Osaka 1‐1 Machikaneyama Toyonaka Osaka 560‐0043 Japan; ^3^ School of Medicine Tokai University Isehara Kanagawa 259–1193 Japan; ^4^ Division of Applied Chemistry Faculty of Engineering Hokkaido University Sapporo Hokkaido 060–8628 Japan; ^5^ Department of Chemistry and Biotechnology Graduate School of Engineering Tottori University 4–101 Koyama‐Minami Tottori 680–8552 Japan; ^6^ Center for Research on Green Sustainable Chemistry Tottori University 4–101 Koyama‐Minami Tottori 680–8552 Japan; ^7^ Interdisciplinary Research Center for Radiation Sciences Institute for Radiation Sciences The University of Osaka 2–4 Yamada‐oka Suita Osaka 565–0871 Japan; ^8^ Center for Advanced Modalities and DDS The University of Osaka 1‐1 Yamadaoka Suita Osaka 565–0871 Japan

**Keywords:** Adjuvant, Drug delivery system, Lipid Nanoparticle, Vaccine

## Abstract

Cancer peptide vaccines harness the host immune system to generate tumor‐specific immune responses, offering potential for treating metastatic cancers and preventing recurrence. However, the limited immunogenicity of peptide antigens restricts their clinical efficacy, necessitating strategies to enhance their potency. One promising approach involves the conjugation of antigens with adjuvants to elicit antigen‐specific immune responses, thereby creating self‐adjuvanting vaccines. In this study, we developed an improved platform for such vaccines. Specifically, the antigenic CH401 peptide was conjugated with the adjuvant (Pam_3_CSK_4_) and formulated into cationic lipid nanoparticles (LNPs) smaller than 100 nm to facilitate uptake by immune cells, with supplemental adjuvants incorporated to modulate immune responses. Notably, the microflow device iLiNP enabled precise, size‐controlled LNP formulation from various components, supporting the systematic evolution of the self‐adjuvanting vaccine platform. These vaccine candidates demonstrated enhanced immunogenicity, enabling precise modulation of immune responses in an antigen‐specific manner. Notably, these vaccines elicited potent immune responses in humanized mouse models. Overall, this strategy represents a next‐generation self‐adjuvanting vaccine platform, capable of both enhancing and fine‐tuning antigen‐specific immune responses, holding great promise for innovative vaccine development.

## Graphical abstract

The self‐adjuvanting vaccine—comprising a covalent conjugate of antigen and adjuvant—was formulated into cationic lipid nanoparticles smaller than 100 nm to facilitate uptake by immune cells, with supplemental adjuvants incorporated to modulate immune responses. Notably, these nanovaccines exhibited potent immunogenicity in humanized mouse models, underscoring their translational potential as next‐generation self‐adjuvanting vaccines.

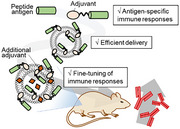

## Introduction

Cancer vaccine therapy harnesses the host immune system to elicit tumor‐specific immunity, offering a promising strategy for treating metastatic cancers and preventing recurrence.^[^ ^1^, ^2^, ^3^, ^4^ ^]^ Among various modalities, peptide‐based vaccines offer distinct advantages.^[^ ^5^, ^6^ ^]^ They target specific epitopes of tumor‐associated antigens (TAAs), thereby minimizing the risk of off‐target adverse effects, and they are cost‐effective to manufacture with well‐defined quality control processes. However, the inherently low immunogenicity of short‐peptide antigens limits their clinical success, underscoring the need for strategies to enhance their immunostimulatory potential.

Physicochemical properties of vaccine formulations critically influence immune recognition and in vivo behavior.^[^ ^5^, ^7^ ^]^ Both carrier proteins, such as keyhole limpet hemocyanin (KLH) and diphtheria toxin (CRM_197_),^[^ ^8^ ^]^ and nanomaterial‐based carriers, including polymers, dendrimers, and self‐assembling materials, have been employed to enhance immunogenicity.^[^ ^5^, ^9^, ^10^, ^11^ ^]^ Among these, lipid nanoparticles (LNPs), which rose to prominence with the coronavirus disease 2019 (COVID‐19) vaccines, have emerged as particularly versatile platforms for delivering peptide antigens.^[^ ^12^, ^13^ ^]^ Their physicochemical properties, including size, surface charge, and stability, can be tuned by lipid composition and formulation parameters. Cationic LNPs approximately 100 nm in diameter are known to promote efficient lymphatic trafficking and cellular uptake.^[^ ^14^, ^15^, ^16^ ^]^ We previously reported that viral replicas formed from self‐assembling virus capsid–derived peptides and encapsulated with cationic lipids generate ∼100 nm cationic nanoparticles, which serve as highly effective carriers for peptide antigens, eliciting robust antibody production.^[^ ^17^ ^]^ Therefore, careful design of vaccine formulation is pivotal for modulating in vivo dynamics and maximizing vaccine efficacy.

TAAs typically require co‐administration of adjuvants to enhance their immunogenicity. Innate immune receptor ligands—many of which are derived from bacterial components—have been extensively investigated as potent adjuvants.^[^ ^18^, ^19^ ^]^ Toll‐like receptor (TLR) agonists, such as Pam_3_CSK_4_ (TLR2/1),^[^ ^20^, ^21^, ^22^, ^23^, ^24^, ^25^ ^]^ monophosphoryl lipid A (MPLA; TLR4),^[^ ^26^, ^27^, ^28^, ^29^ ^]^ and CpG motif (TLR9),^[^ ^30^ ^]^ as well as glycolipids like α‐galactosylceramide (α‐GalCer), which engage cluster of differentiation 1d (CD1d),^[^ ^31^, ^32^ ^]^ have demonstrated potent adjuvant effects in both preclinical and clinical settings. Furthermore, LNP formulations of innate immune receptor ligands have been explored to enhance their immunostimulatory activities. Among the cationic adjuvant formulation (CAF) families, CAF01—containing trehalose 6,6′‐dibehenate (TDB)—and CAF09—containing polyinosinic‐polycytidylic acid [Poly(I:C)]—are under clinical investigation.^[^ ^33^ ^]^ The adjuvant system (AS) families represent modular adjuvant platforms, including AS01 (a liposomal formulation containing MPLA and QS‐21), AS03 (an oil‐in‐water emulsion with squalene and α‐tocopherol), and AS04 (MPLA adsorbed onto aluminum hydroxide), which have been licensed for human use.^[^ ^34^ ^]^ Importantly, activation of multiple innate pathways can synergistically amplify immune responses,^[^ ^35^, ^36^ ^]^ making combination adjuvant strategies a compelling approach.^[^ ^37^, ^38^, ^39^, ^40^ ^]^ However, these adjuvants may also induce systemic inflammation, necessitating strategies that balance potency with safety.

A promising approach to address this limitation is the use of self‐adjuvanting vaccine strategies, in which antigens and innate immune ligands are covalently linked.^[^ ^41^, ^42^, ^43^ ^]^ This design ensures the co‐delivery of both components to antigen‐presenting cells (APCs), thereby inducing antigen‐specific immunity while minimizing nonspecific inflammation. Since the early work by Boons et al.,^[^ ^44^ ^]^ Pam_3_CSK_4_ has been widely used in self‐adjuvanting conjugate vaccines.^[^ ^45^, ^46^, ^47^, ^48^, ^49^ ^]^ Other innate immune ligands, including α‐GalCer^[^ ^50^, ^51^, ^52^, ^53^ ^]^ and MPLA,^[^ ^54^, ^55^, ^56^, ^57^ ^]^ have also been employed. Furthermore, co‐loading antigen and adjuvant into a single particulate carrier has been shown to confer self‐adjuvanting properties.^[^ ^58^, ^59^, ^60^ ^]^

**Figure 1:**
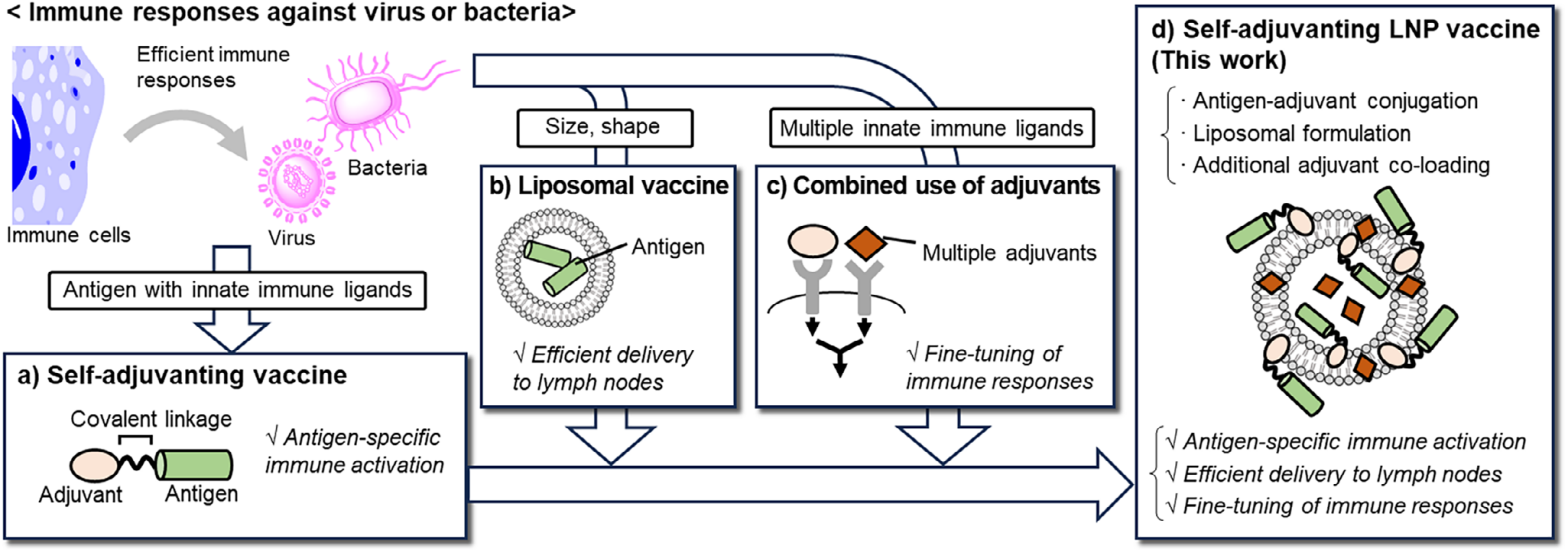
Systematic strategy employed in this study for developing next‐generation self‐adjuvanting vaccines, inspired by natural immune response to infectious agents. a) Self‐adjuvanting vaccine, b) liposomal vaccine, c) combined use of adjuvants, and d) self‐adjuvanting lipid nanoparticles (LNP) vaccine (this study).

In this study, we aimed to advance the design of self‐adjuvanting cancer vaccines by incorporating pathogen‐inspired features into a modular nanoparticle platform (Figure 1). Specifically, we integrated: i) co‐delivery of antigen and adjuvant to the same immune cells (Figure 1a); ii) pathogen‐like nanoscale architecture to enhance immunological recognition (Figure 1b); and iii) engagement of multiple innate immune pathways to drive synergistic immune activation (Figure 1c). To implement this design, we developed self‐adjuvanting LNP vaccines that integrate all of these principles (Figure 1d); the antigen‐adjuvant conjugated vaccine (self‐adjuvanting vaccine) was loaded into LNPs to improve delivery efficacy, and supplementary adjuvants were co‐loaded to enable fine‐tuning of the immune responses via multiple‐adjuvant synergistic effects. Based on natural immune responses to pathogens, this study systematically integrates essential components required for effective immune activation.

**Figure 2:**
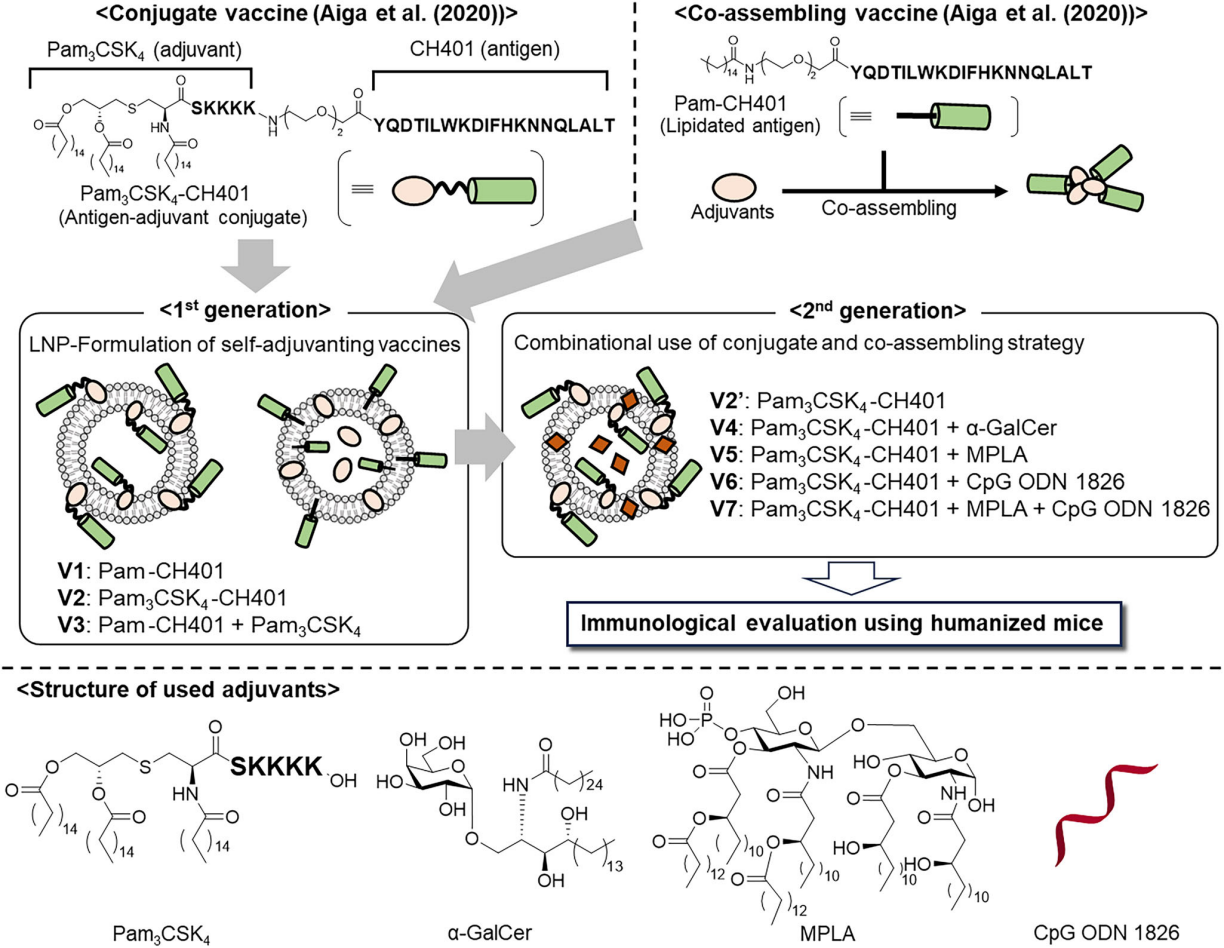
Overview of the study design, composition of each vaccine formulation, and chemical structures of Pam_3_CSK_4_–CH401 (a covalent conjugate of Pam_3_CSK_4_ and CH401) and Pam–CH401 (palmitoylated CH401), along with the adjuvants used.

To implement this concept, building upon our previous work on a self‐adjuvanting conjugate vaccine comprising the human epidermal growth factor receptor 2 (HER2)‐derived CH401 peptide antigen^[^ ^61^, ^62^ ^]^ and Pam_3_CSK_4_ (Pam_3_CSK_4_–CH401),^[^ ^17^, ^60^, ^63^ ^]^ as well as co‐assembling vaccines of lipidated CH401 (palmitoylated CH401: Pam–CH401) with various lipophilic adjuvants,^[^ ^60^ ^]^ we systematically optimized LNP‐based formulations to enhance both the efficacy and tunability of immune responses (Figure 2). Our first‐generation vaccines (**V1**–**V3**) utilized LNP‐encapsulated Pam_3_CSK_4_–CH401, which promoted strong class switching and high IgG titers. To further refine immune modulation, we engineered second‐generation formulations (**V4**–**V7**) by co‐loading additional adjuvants onto/into the LNPs, enabling precise immune modulation through multi‐adjuvant synergy. Specifically, simultaneous activation of innate immune pathways not only enhanced antibody production but also enabled controlled polarization of immune responses—such as modulation of the balance between T helper type (Th1) and type (Th2) responses—depending on the adjuvant combinations employed.

An additional key aspect of this study is the immunological evaluation using humanized mice (PBL‐NOG‐hIL‐4‐Tg). These mice possess a more clinically relevant immune landscape than conventional wild‐type strains, such as BALB/c, whose murine immune responses often diverge from those of humans. Notably, the vaccine candidates elicited both robust antibody production and cytotoxic T‐cell activation in this humanized model, highlighting their capacity to orchestrate multifaceted adaptive immune responses and underscoring the translational potential of our approach.

Despite the considerable promise of self‐adjuvanting vaccines, the formulation of these relatively new vaccine materials remains underexplored. In this study, we developed a precise, size‐controlled LNP formulation of self‐adjuvanting vaccines co‐loaded with supplementary adjuvants, enabling systematic modulation of immune responses. This study demonstrates that the rational integration of structural and immunological design principles—derived from pathogen recognition—can generate next‐generation cancer vaccines with enhanced potency and programmable immune profiles. Collectively, this platform achieves both functional enhancement through LNP formulation and precise immune modulation via multi‐adjuvant synergy, representing a significant advancement in the engineering of self‐adjuvanting cancer vaccines.

## Results

### Preparation and Evaluation of First‐Generation Self‐Adjuvanting LNP Vaccines

**Figure 3:**
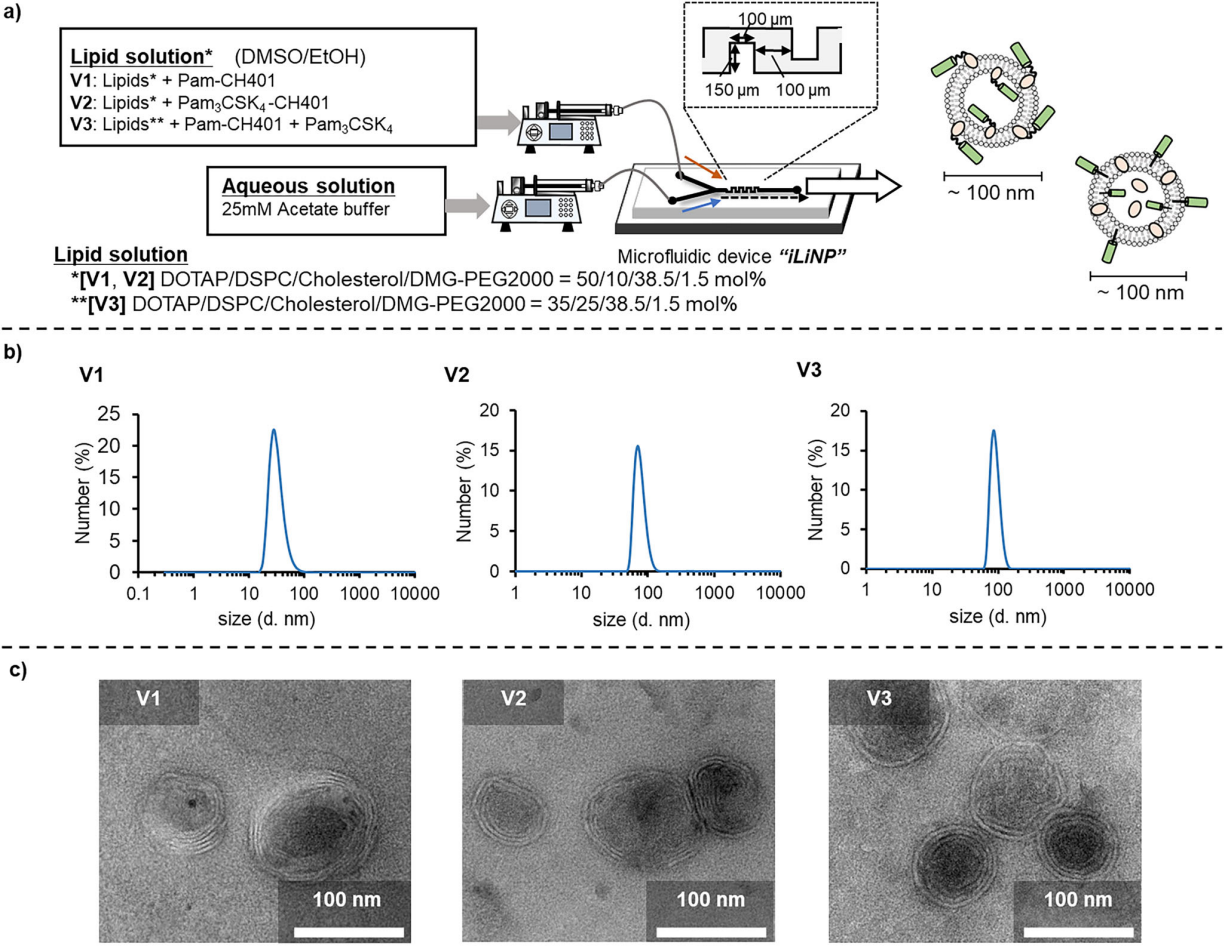
Preparation of first‐generation self‐adjuvanting LNP vaccines **V1**–**V3**. a) Preparation method for **V1**–**V3**. b) Dynamic light scattering (DLS)‐based particle size distribution of **V1**–**V3**. c) Transmission electron microscopy (TEM) images of **V1**–**V3**.

We designed first‐generation self‐adjuvanting LNP vaccines, **V2** and **V3**, by nanoparticulating our previously reported CH401‐based conjugate and co‐assembling self‐adjuvanting vaccines. The composition of each vaccine is shown in Figure 3. **V1** consisted of LNPs incorporating only the CH401 antigen (Pam–CH401) without any adjuvants (control), whereas **V2** incorporated the Pam_3_CSK_4_‐conjugated CH401 (Pam_3_CSK_4_–CH401)^[^ ^60^ ^]^ into LNPs. **V3** was prepared by co‐loading Pam_3_CSK_4_ with Pam‐CH401. For LNP formulation, we used the cationic lipid 1,2‐dioleoyl‐3‐trimethylammonium propane (DOTAP) as the main component to generate cationic particles, which are favorable for uptake by immune cells. Polyethylene glycol–modified lipid (1,2‐dimyristoyl‐rac‐glycero‐methoxypolyethylene glycol‐2000; DMG‐PEG2000) was also included to enhance the metabolic stability of the particles.^[^ ^64^ ^]^ The CH401 peptide antigen was lipidated with Pam or Pam_3_CSK_4_, and the resulting Pam–CH401 (**V1**, **V3**) and Pam_3_CSK_4_–CH401 (**V2**) were expected to be incorporated into the lipid bilayer, displaying the CH401 antigen on the LNP surface to enhance immune cell recognition.

To formulate these components into sub‐100 nm spherical particles, which enable efficient delivery to the lymph nodes, we utilized the microfluidic iLiNP device (Figure 3a).^[^ ^65^, ^66^ ^]^ The iLiNP device efficiently mixes lipid and aqueous solutions within a zigzag‐shaped microchannel, allowing reproducible preparation of LNPs with precise control over particle size by adjusting the flow rate. Lipid solutions containing a 4 mM lipid mixture (DOTAP/1,2‐distearoyl‐sn‐glycero‐3‐phosphocholine [DSPC]/cholesterol/DMG‐PEG2000 = 50/10/38.5/1.5 molar ratio for **V1** and **V2**, or 35/25/38.5/1.5 molar ratio for **V3**; Because **V3** did not form homogeneous LNPs with 50 mol% DOTAP, the proportion of DOTAP was adjusted.) in ethanol (EtOH)/dimethyl sulfoxide (DMSO), together with antigen and adjuvant components (**V1**: 0.04 mM Pam–CH401, **V2**: 0.04 mM Pam_3_CSK_4_–CH401, **V3**: 0.04 mM Pam–CH401 and Pam_3_CSK_4_), and 25 mM acetate buffer (pH = 4.0) were pumped at flow rates of 125 and 375 µL min^−1^, respectively. The resulting mixtures were subsequently dialyzed to obtain the final vaccine formulations **V1**–**V3**.

The size and morphology of the LNP vaccines **V1**–**V3** were characterized using dynamic light scattering (DLS) and transmission electron microscopy (TEM). DLS analysis revealed that all vaccine formulations (**V1**–**V3**) exhibited particle sizes below 100 nm with high uniformity (Figures 3b and S1–S4). As expected, the *ζ*‐potentials of **V1**–**V3** ranged from +20 to +30 mV, confirming their cationic nature (Figure S11). Although **V3** contained less DOTAP than **V1** and **V2**, its comparable ζ‐potential may suggest that this difference did not substantially influence the immune responses. TEM imaging revealed that all **V1**–**V3** exhibited spherical, multilamellar, liposome‐like structures approximately 100 nm in diameter (Figures 3c and S21–S23). The particle sizes of **V1**–**V3** remained largely stable during dialysis at 4 °C for one week (Figures S13–S15), indicating good colloidal stability, although **V2** showed higher size dispersity. Incorporation of the respective antigens and adjuvants into LNPs was verified by liquid chromatography–mass spectrometry (LC–MS); 70%–90% of Pam–CH401 (**V1** and **V3**) and 40%–50% of Pam_3_CSK_4_–CH401 (**V2**) were successfully incorporated, and most of Pam_3_CSK_4_ (**V3**) was also incorporated, although exact quantification was not performed. Notably, the iLiNP device enabled efficient and highly reproducible formulation of all vaccine candidates **V1**–**V3**, achieving the intended particle size and morphology through a simple operational process.

**Figure 4:**
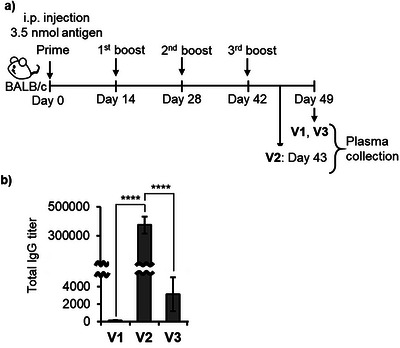
Immunological evaluation of **V1–V3**. a) Schematic of the vaccination protocol. b) Anti‐CH401 IgG antibody titers following four immunizations with **V1–V3**. Data represent results from five independent experiments (*n* = 5), and error bars indicate the standard error of the mean. One‐way ANOVA followed by Tukey's test using GraphPad Prism 9: **** *p* < 0.0001.

LNP‐based vaccine candidates **V1**–**V3** in phosphate‐buffered saline (PBS) were intraperitoneally administered to BALB/c mice (*n* = 5) biweekly on four separate occasions (Days 0, 14, 28, and 42) (Figure 4a). For each immunization, **V1**–**V3** were delivered at a dose corresponding to 35 µM CH401 (Pam‐CH401 or Pam_3_CSK_4_–CH401). Blood samples were collected one week after each immunization (Days 7, 21, 35, and 49). During the immunization process, one mouse each from the **V1** and **V2** groups either died or became moribund (see Supporting Information); data from these mice were excluded from subsequent antibody titer and splenocyte analyses. No overt clinical symptoms were observed in the remaining mice.

Anti‐CH401 antibody titers in mice immunized with **V1**–**V3** were quantified (Figures 4b and S29–S35). As expected, the adjuvant‐co‐loaded formulations **V2** and **V3** induced higher IgG titers than **V1**, confirming their self‐adjuvanting properties. Notably, **V2** elicited significantly higher titers than **V3**, underscoring the critical role of covalent conjugation between the antigen and adjuvant in producing a highly stable complex. Evaluation of cytokine secretion from splenocytes revealed that **V3** induced a distinct cytokine profile, whereas **V2** did not induce excessive cytokine secretion, further supporting its superior capacity for antigen‐specific immune induction (Figure S42). In our previous study, Pam_3_CSK_4_–CH401 formed an oil‐in‐water emulsion in PBS, yielding spherical aggregates of approximately 100 nm that effectively induced IgG production.^[^ ^60^ ^]^ In contrast, incorporation of Pam_3_CSK_4_–CH401 into uniform cationic LNPs (**V2**) maintained the high IgG response while significantly reducing IgM titers, indicating that this LNP formulation promotes efficient class switching from IgM to IgG (Figure S29). Collectively, these findings demonstrate that LNP formulation enhances the immunological performance of self‐adjuvanting vaccines and, in particular, that LNP‐based delivery of the covalent conjugate vaccine enables robust, antigen‐specific immune responses.

### Preparation and Evaluation of Second‐Generation Self‐Adjuvanting LNP Vaccines

Building on these promising results, we designed a second‐generation self‐adjuvanting LNP vaccine (Figure 2). In the preceding experiments, **V2** incorporating covalent conjugate Pam_3_CSK_4_–CH401 into LNPs—effectively elicited antigen‐specific immune responses. Additionally, the co‐loaded adjuvant Pam_3_CSK_4_ in **V3** enhanced immune responses compared with **V1**. Based on these findings, we co‐loaded additional adjuvants into **V2** to fine‐tune its antigen‐specific immune response profile. Specifically, α‐GalCer (CD1d ligand), MPLA (TLR4 ligand), and CpG ODN 1826 (TLR9 ligand) were incorporated into Pam_3_CSK_4_–CH401‐containing LNPs to generate **V4**–**V6**, respectively. Furthermore, we investigated the potential synergistic effects of multiple innate immune ligands.^[^ ^36^ ^]^ Given that Pam_3_CSK_4_, CpG ODN 1826, and MPLA have been reported to produce a Th1‐biased synergistic effect,^[^ ^40^ ^]^ we co‐loaded both MPLA and CpG ODN 1826, along with Pam_3_CSK_4_–CH401, into **V7**. However, the synergistic adjuvant effects of self‐adjuvanting vaccines have rarely been investigated. We anticipate that this approach will enable precise immune modulation and facilitate the design of antigen‐specific immune responses.

The newly designed **V4**–**V7** formulations were prepared using the iLiNP device. Lipid solutions composed of a 4 mM lipid mixture (DOTAP/DSPC/cholesterol/DMG‐PEG2000 = 50/10/38.5/1.5 molar ratio) dissolved in EtOH/DMSO, along with Pam_3_CSK_4_–CH401 and lipophilic adjuvants (**V4**: 0.04 mM Pam_3_CSK_4_–CH401 and α‐GalCer; **V5**: 0.04 mM Pam_3_CSK_4_–CH401 and MPLA; **V6**: 0.04 mM Pam_3_CSK_4_–CH401; **V7**: Pam_3_CSK_4_–CH401 and MPLA), and 25 mM acetate buffer (pH = 4.0) (**V6** and **V7**: containing 35.3 µg mL^−1^ CpG ODN 1826, nitrogen/phosphate [*N*/*P*] ratio: 6) were pumped at flow rates of 25 and 75 µL min^−1^, respectively (flow‐rate ratio = 3). Compared with the first‐generation vaccines (**V1**–**V3**), the flow rate was decreased to adjust the particle size to a similar range. The resulting solutions were dialyzed to obtain **V4**–**V7**. As intended, all **V4**–**V7** formulations, despite their differing components, formed spherical, cationic particles approximately 100 nm in diameter (DLS: Figures S5–S9; *ζ*‐potential: Figure S12; TEM: Figures S24–S28), demonstrating the robustness of LNP preparation using the iLiNP device. The particle sizes of **V4**–**V7** remained consistent after storage at 4 °C for one month (Figures S16–S19), confirming their stability. The incorporation of the respective antigens and adjuvants into the LNPs was confirmed using LC–MS or the RiboGreen assay. Approximately 40%–50% of Pam_3_CSK_4_–CH401 was successfully incorporated, along with the insertion of each lipophilic adjuvant (see Supplementary Information, preparation of each vaccine formulation) and encapsulation of CpG ODN 1826 (Table S1). Notably, the iLiNP device facilitated the formulation of all vaccine candidates **V4**–**V7** with comparable physical properties (40–100 nm size, spherical, cationic, and multilamellar structures), thereby permitting meaningful comparisons among the respective components while maintaining their delivery characteristics.

**Figure 5:**
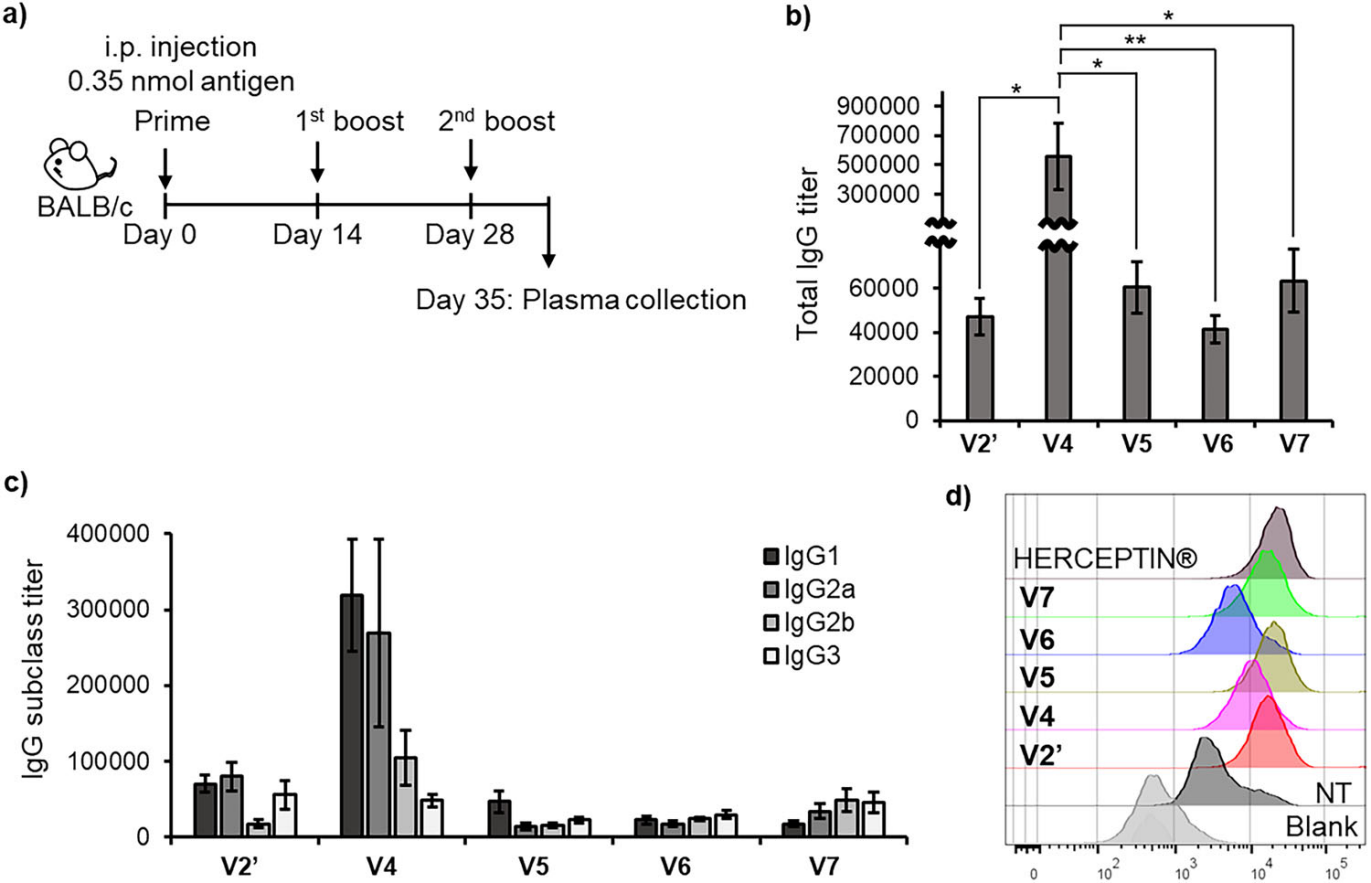
Immunological evaluation of **V2’** and **V4**–**V7**. a) Schematic of the vaccination protocol. b) Anti‐CH401 IgG antibody titers after three immunizations with **V2’** or **V4**–**V7**. c) Anti‐CH401 IgG antibody subtypes in plasma from mice vaccinated with **V2’** or **V4**–**V7**. Data represent results from five independent experiments (*n* = 5), and error bars indicate the standard error of the mean. One‐way ANOVA followed by Tukey's test using GraphPad Prism 9: * *p* < 0.05; ** *p* < 0.005. d) Flow cytometric analysis of binding between plasma samples (1:50 dilution) collected on Day 49 from mice immunized with **V2’** or **V4**–**V7** and the human breast cancer cell line BT‐474. Alexa Fluor 488‐conjugated goat anti‐mouse IgG antibody (1:50 dilution) was used as a secondary antibody for staining. Blank: treated with the secondary antibody only. NT: treated with plasma samples collected before vaccination, followed by the secondary antibody; **V2’** and **V4**–**V7**: pooled plasma from each group (*n* = 5) of mice.

BALB/c mice (*n* = 5) were immunized with **V4**–**V7** (Figure 5a). Given the partial toxicity observed during the administration of **V1** and **V2**, both the dose and frequency were reduced for **V4**–**V7** (**V1**–**V3**: 3.5 nmol/dose, 4 doses; **V4**–**V7**: 0.35 nmol/dose, 3 doses). As a control, a modified **V2** (denoted as **V2'**) was prepared; **V2'** was formulated using the iLiNP device at the same flow rate as **V4**–**V7**, adjusted to one‐tenth the concentration of **V2**, and exhibited a particle size comparable to that of **V2** (DLS: Figure S10; DLS after storage at 4 °C for one month: Figure S20; TEM: Figure S28), with its dose and frequency adjusted to match those of **V4**–**V7**. Under this protocol, no signs of debilitation were observed in any mice.

The anti‐CH401 antibody titers induced by **V2'** and **V4**–**V7** highlighted the effects of the co‐loaded adjuvants (Figures 5b and S36–S41). **V4**, in which α‐GalCer was co‐loaded with Pam_3_CSK_4_–CH401, exhibited a significantly higher IgG antibody titer, likely owing to the synergistic activation of innate immunity by α‐GalCer and Pam_3_CSK_4_. Moreover, subclass analysis of the induced anti‐CH401 IgG antibodies revealed significant differences in immune responses among **V2'**, **V4**, **V5**, **V6**, and **V7**, emphasizing the synergistic effect of multiple adjuvants (Figure 5c). When evaluating the IgG2a/IgG1 ratio, an indicator of Th1/Th2 bias, **V2'**, which lacked co‐loaded adjuvants, exhibited a slightly more IgG2a‐predominant response (IgG2a/IgG1 = 1.4). In contrast, additional adjuvant co‐loading altered the IgG subclass distribution. Compared to **V2'**, **V4**, **V5**, and **V6** showed a more IgG1‐biased antibody production (IgG2a/IgG1: **V4** = 0.7, **V5** = 0.5, **V6** = 1.1), whereas **V7** demonstrated a more IgG2a‐biased response (IgG2a/IgG1 = 2.0). Notably, **V7**, co‐adjuvanted with MPLA and CpG ODN 1826, both known to induce Th1‐type immune responses^[^ ^18^, ^40^, ^67^, ^68^ ^]^—predominantly stimulated IgG2a antibody production, indicating robust Th1‐biased immune responses as expected. Given that Th1‐skewed immune responses are associated with the activation of cytotoxic T cells and that IgG2a has been reported to induce strong cytotoxic activity through complement‐dependent cytotoxicity (CDC) and antibody‐dependent cellular cytotoxicity (ADCC) in mice,^[^ ^69^ ^]^ **V7** shows promise for eliciting potent anticancer immune responses. Overall, incorporating supplementary adjuvants into conjugate vaccine (Pam_3_CSK_4_–CH401)‐loaded LNPs enabled the targeted modulation of immune responses.

Furthermore, flow cytometric analysis confirmed that antibodies induced by **V2'** and **V4**–**V7** recognized the HER2‐expressing breast cancer cell line BT474 (Figure 5d). Notably, **V7** generated antibodies with high affinity, comparable to that of Herceptin, the clinically approved anti‐HER2 antibody.

### Validation of Immune Responses Using Humanized Mice

**Figure 6:**
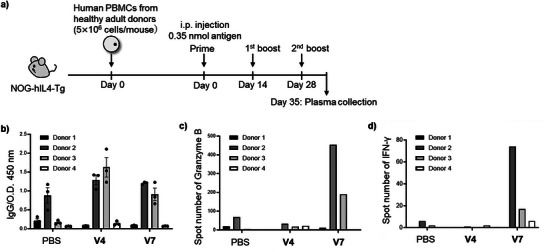
Immunological evaluation of **V4** and **V7** in humanized mice. a) Schematic representation of humanized mouse preparation and vaccination protocol. b) Anti‐CH401 IgG antibody titers after three immunizations with **V4** or **V7** (1:125 dilution). Each dot represents an independent experiment (*n* = 3). Bars represent the mean of three experiments, and error bars represent the standard error of the mean. (c, d) ELISpot analysis of splenocytes harvested from mice vaccinated with **V4** or **V7**. Spot counts of c) granzyme B and d) Interferon (IFN)‐γ secretion by splenocytes following stimulation with CH401‐MAP.

We next administered **V4**, which induces a high IgG antibody titer, and **V7**, which elicits characteristic Th1‐type immune responses, to humanized mice^[^ ^70^ ^]^ to evaluate their functionality within the human immune system. We selected the humanized NOG‐hIL4‐Tg mouse system^[^ ^71^ ^]^ as it allows efficient engraftment of human T and B cells and supports antigen‐specific IgG production through the transplantation of human peripheral blood mononuclear cells (PBMCs) into human‐IL‐4‐gene‐transfected NOD/Shi‐scid IL‐2RγKO (NOG) (PBL‐NOG‐hIL‐4‐Tg) mice. PBMCs were obtained from four healthy donors (Donors 1–4), who were recruited following informed consent. The PBMCs were prepared via density‐gradient centrifugation and intravenously transplanted into recipient mice. These mice were then immunized with **V4** and **V7** at a dose of 0.35 nmol of Pam_3_CSK_4_–CH401 every two weeks, for a total of three administrations (Figure 6a), with a PBS‐injected group included as a control.

Spleen cells were collected on Day 35, and engraftment efficiency as well as immune cell profiles were examined. Human cell engraftment varied in a donor‐dependent manner across the three treatment groups (Figure S44). PBMCs derived from Donor 2 exhibited the highest engraftment, with T, B, natural killer (NK), and NKT cells all confirmed (Figures S45–S48). The gating strategy is illustrated in Figures S49–S57. In contrast, PBMCs from other donors showed lower levels of human leukocyte engraftment. Notably, the proportion of exhausted T cells (CD25^+^PD‐1^+^ cells) remained low in all groups (Figures S47c and S47e).

Analysis of plasma antibody titers revealed increased levels of IgG and IgM in **V4**‐ and **V7**‐immunized mice transplanted with PBMCs from Donors 2 and 3 (Figures 6b and S43), whereas PBS‐injected controls produced only negligible anti‐CH401 antibodies. These results suggest that both **V4** and **V7** function effectively within the human immune system to induce anti‐CH401 antibodies. Notably, **V4** generated a high IgG titer, indicating its potential as a promising vaccine candidate with robust antibody‐inducing activity in both humanized and BALB/c mice, despite a B cell count below 1 × 10^6^. In contrast, mice transplanted with PBMCs from Donors 1 and 4 showed no significant anti‐CH401 antibody production, likely attributed to low B cell engraftment (Figures S45 and S49–S51) and the absence of clonal cells capable of recognizing the CH401 epitope. Notably, although B cell engraftment was higher in **V7**‐treated mice than in **V4**‐treated mice for Donors 2 and 3, the resulting antibody titers were lower. This suggests that **V4** efficiently stimulated antibody production even from fewer B cells, whereas **V7** may have promoted B cell proliferation and engraftment but was less effective in promoting antibody production. These findings confirm that the potent antibody‐inducing capacity of **V4** observed in murine models is also evident within the human immune system.

To further investigate immune responses and assess the effects of co‐loaded adjuvants, we performed cytokine profiling. Although antibody production is typically associated with Th2‐skewed responses, Th1‐biased environments are essential for cytotoxic activity. **V4** was formulated with α‐GalCer, which enhances both Th1‐ and Th2‐associated cytokines, whereas **V7** contained MPLA and CpG ODN 1826, both known to promote Th1 polarization. Therefore, we hypothesized that **V7** would elicit a stronger cytotoxic response. Given the limited number of transplanted cells, we evaluated cytokine production using an enzyme‐linked immunospot (ELISpot) assay, which allowed the detection of responses at the single‐cell level (Figures 6c,d, S58, and S59). Splenocytes from **V7**‐vaccinated mice secreted significantly higher levels of granzyme B and interferon (IFN)‐γ in response to stimulation with CH401. In particular, granzyme B is closely associated with cytotoxic T cell activity, and its elevated secretion indicates effective stimulation of cellular immunity by **V7**. As cellular immunity is critical in cancer immunotherapy, these findings highlight **V7** as a promising vaccine candidate. Additionally, **V7** induced production of IFN‐γ, a Th1‐associated cytokine, consistent with findings from mouse immunization experiments in which **V7** promoted Th1‐biased immune responses leading to predominant IgG2a antibody production (Figure 5c). Detailed cytokine profiling revealed that **V4** elicited responses similar to those of the PBS‐treated control group, whereas **V7** induced markedly different patterns. Specifically, **V7** increased secretion of IFN‐γ, interleukin (IL)‐10, IL‐5, IL‐13, and IL‐22, while decreasing that of IL‐2 (Figure S60). These findings suggest that the three adjuvants in **V7**—Pam_3_CSK_4_, MPLA, and CpG ODN 1826—function synergistically to induce the Th1 cytokine IFN‐γ, the Th2 cytokines IL‐5 and IL‐13, the immunosuppressive cytokine IL‐10, and the inflammatory cytokine IL‐22, without elevating Th17‐associated cytokines or other pro‐inflammatory cytokines such as IL‐6 and tumor necrosis factor (TNF)‐α.

Overall, experiments using humanized mice demonstrated that **V4** and **V7** elicited distinct immune responses consistent with their respective adjuvant compositions. Although NOG mice transplanted with a limited number of human cells possessed a restricted repertoire for quantitative evaluation of antibody production—allowing only qualitative assessment of antibody titers—our results demonstrate that both **V4** and **V7** successfully induced CH401‐specific immune responses within the human immune system, consistent with their intended mechanisms of T and B cell activation.

## Discussion

In this study, we advanced next‐generation vaccine design by enhancing the functionality of self‐adjuvanting vaccines through the use of LNPs as a delivery platform. The self‐adjuvanting approach—based on the covalent linkage between antigen and adjuvant—has been demonstrated to elicit potent, antigen‐specific immune responses.^[^ ^41^, ^42^, ^43^ ^]^ In particular, lipophilic adjuvants have shown notable success in this context, serving not only to activate immune responses but also to promote the formation of oil‐in‐water emulsions, thereby improving the metabolic stability and cellular uptake of vaccine components.^[^ ^72^, ^73^ ^]^ These observations underscore the importance of formulation in determining the performance of self‐adjuvanting vaccines. Furthermore, incorporating multiple adjuvants offers a promising route to achieving robust immune enhancement and precise immune modulation, including the controlled regulation of inflammatory responses. However, the synthesis of covalently conjugated vaccines incorporating multiple adjuvants is technically complex and economically demanding, highlighting the need for alternative strategies. In this study, we addressed these challenges by formulating a covalent conjugate of the CH401 antigen and the Pam_3_CSK_4_ adjuvant into LNPs, while simultaneously co‐loading supplementary adjuvants. Although previous applications of LNPs for lipophilic self‐adjuvanting vaccines have largely focused on overcoming water‐solubility limitations, our approach leveraged LNP formulation as an active means of enhancing functional performance. Beyond improving solubility, LNPs also provide a versatile platform that enables synergistic immune activation through the coordinated codelivery of multiple adjuvants to the same immune cells. Consistent with this design principle, LNP‑formulated Pam_3_CSK_4_–CH401 (**V2**) showed greater potency than its non‑LNP counterpart, and the incorporation of additional adjuvants (**V4**–**V7**) further allowed targeted modulation of immune responses.

The physicochemical properties of the delivery vehicle are pivotal determinants of vaccine efficacy. Particle size is particularly critical; particles <10 nm can readily enter blood capillaries, whereas those >100 nm are unable to drain into lymphatic vessels from the interstitium. Consequently, the 10–100 nm range is considered optimal for lymph node targeting. Additional parameters—including a cationic surface charge and multivalent antigen presentation—further promote immune cell uptake.^[^ ^14^, ^15^, ^16^ ^]^ Based on these principles, we employed iLiNP technology to generate cationic LNPs within the optimal size range. The robust shape‑forming capability of iLiNPs enabled the co‐loading of multiple components without compromising particle size. As expected, LNP formulation enhanced vaccine efficacy: LNP‑formulated **V2** promoted more efficient Ig class switching than non‑LNP‑formulated Pam_3_CSK_4_–CH401, while **V3**—co‑loading Pam_3_CSK_4_ with Pam‑CH401—elicited higher antibody titers than corresponding non‑spherical co‑aggregates.^[^ ^60^ ^]^ Collectively, these findings validate that the rational formulation design can substantially improve the performance of self‑adjuvanting vaccines.

Notably, the adjuvant co‐loaded with Pam_3_CSK_4_‐CH401 in **V4**–**V7** modulated immune responses in an antigen‐specific manner. While the synergistic effects of multi‐adjuvant combinations are well established,^[^ ^35^, ^36^ ^]^ achieving antigen‐specific synergy in vaccine development remains a challenge. Multi‐adjuvant‐conjugated self‐adjuvanting vaccines have been reported,^[^ ^74^, ^75^, ^76^ ^]^ but they require labor‐intensive synthetic procedures. In contrast, our approach—using a covalent antigen–adjuvant conjugate as an immunological “anchor” and incorporating supplementary adjuvants into LNPs—achieved antigen‑specific synergy without additional synthetic complexity. Mechanistically, Pam_3_CSK_4_ induces a Th2‐biased immune response,^[^ ^77^, ^78^ ^]^ while α‐GalCer promotes both Th1 and Th2 cytokine production,^[^ ^79^, ^80^ ^]^ and MPLA or CpG ODN1826 promote Th1‐biased immunity.^[^ ^18^, ^40^, ^67^, ^68^ ^]^ In this study, α‐GalCer‐co‐loaded **V4** enhanced CD4^+^ Th2 activation and antibody production, whereas **V7**—co‑loaded with MPLA and CpG ODN1826—shifted the Th1/Th2 balance toward Th1‐dominance, yielding predominantly IgG2a antibodies. Therefore, covalent linkage antigen–adjuvant linkage ensured robust antigen‐specific immunity, while selective co‐loading adjuvants enabled fine‐tuning of response quality. This platform offers a practical route to engineer the qualitative profile of antigen‐specific immunity in self‐adjuvanting vaccines.

Crucially, the vaccine formulations developed in this study effectively activated human immune cells, representing a significant step toward clinical translation. Species‐specific differences in major histocompatibility complex (MHC) restriction and innate receptor recognition^[^ ^81^ ^]^ often hinder direct extrapolation of murine data to humans. By contrast, the PBL‐NOG‐hIL‐4‐Tg mouse model captures intrinsic donor immune variability and facilitates concurrent evaluation of human leukocyte antigen (HLA)–T cell receptor (TCR) interactions and adjuvant efficacy, providing a more predictive platform for human vaccine assessment. As expected, we observed substantial inter‐individual variation in immune responses between the **V4** and **V7** treatment groups, highlighting the utility of humanized mouse models for detecting donor‐dependent immune responsiveness—a critical factor in personalized medicine. Consequently, these vaccines may induce heterogenous outcomes reflecting recipients’ immunological backgrounds. These findings underscore the value of the PBL‐NOG‐hIL‐4‐Tg model in future vaccine and personalized immunotherapy development.

## Conclusion

We developed and evaluated LNP‐formulated self‐adjuvanting vaccines, yielding two key findings: i) covalent conjugation of antigen and adjuvant within LNPs significantly enhances antibody production compared to separate loading (**V2** versus **V3**); and ii) incorporation of additional adjuvants into these LNPs facilitates synergistic, antigen‐specific modulation of immune responses in vivo (**V2** versus **V4**–**V7**).

The immune system is evolutionarily tuned to detect nanoscale pathogens presenting antigens alongside multiple innate immune ligands. By mimicking this natural architecture, our vaccine platform effectively engages the human immune system. Collectively, our findings establish a foundation for innovative vaccine designs that precisely regulate antigen‐specific immune responses.

## Supporting Information

The authors have cited additional references within the Supporting Information.^[^ ^82^, ^83^ ^]^

## Conflict of Interests

The authors declare no conflict of interest.

## Supporting information



Supporting Information

## Data Availability

The data that support the findings of this study are available in the Supporting Information of this article.
