# Supplementary material for: Programmable Antigen‐Specific Immunity via Self‐Adjuvanting Nanovaccines Co‐Delivering Immune Modulators
Source: Angew Chem Int Ed Engl. 2025 Dec 17;65(11):e20474. doi: 10.1002/anie.202520474 (PMC12970494; doi:10.1002/anie.202520474)
Supplement: Supplementary file 1 — Supporting Information [file ANIE-65-e20474-s001.pdf]

Supporting information

Programmable Antigen-Specific Immunity via Self-Adjuvanting Nanovaccines Co-Delivering Immune Modulators

Keita Ito,[a] Yoshiyuki Manabe,*[a][b] Shino Ohshima,[c] Masatoshi Maeki,[d] Manabu Tokeshi,[d] Hiroshi Inaba,[e][f] Kazunori Matsuura,[e][f] Kazuya Kabayama,[a][b][g] Yoshie Kametani,*[c] and Koichi Fukase,*[a][b][g][h]

[a] Dr. K. Ito, Dr. Y. Manabe, Prof. K. Kabayama, Prof. K. Fukase
Department of Chemistry, Graduate School of Science, The University of Osaka
1-1 Machikaneyama, Toyonaka, Osaka 560-0043, Japan
manabey12@chem.sci.osaka-u.ac.jp, koichi@chem.sci.osaka-u.ac.jp

[b] Dr. Y. Manabe, Dr. K. Kabayama, Prof. K. Fukase
Forefront Research Center, The University of Osaka
1-1 Machikaneyama, Toyonaka, Osaka 560-0043, Japan

[c] S. Ohshima, Prof. Y. Kametani
School of Medicine, Tokai University
Isehara, Kanagawa 259-1193, Japan
y-kametn@tokai.ac.jp

[d] Dr. M. Maeki, Prof. M. Tokeshi
Division of Applied Chemistry, Faculty of Engineering, Hokkaido University
Sapporo, Hokkaido 060-8628, Japan

[e] Dr. H. Inaba, Prof. K. Matsuura
Department of Chemistry and Biotechnology, Graduate School of Engineering, Tottori University
4-101 Koyama-Minami, Tottori 680-8552, Japan

[f] Dr. H. Inaba, Prof. K. Matsuura
Center for Research on Green Sustainable Chemistry, Tottori University
4-101 Koyama-Minami, Tottori 680-8552, Japan

[g] Dr. K. Kabayama, Prof. K. Fukase
Interdisciplinary Research Center for Radiation Sciences, Institute for Radiation Sciences, The University of Osaka
2-4 Yamada-oka, Suita, Osaka 565-0871, Japan

[h] Prof. K. Fukase
Center for Advanced Modalities and DDS, The University of Osaka
1-1 Yamadaoka, Suita, Osaka 565-0871, Japan

Table of Contents

1. Materials ... 3

1-1. Synthesis of Materials ... 3

1-2. Protocol for the preparation of V1-V7 ... 3

1-3. Ribogreen assay ... 8

1-4. DLS analysis ... 9

1-5. ζ-Potential ... 13

1-6. Nanoparticle stability ... 14

1-7. TEM analysis ... 16

2. Mice immunization ... 19

3. ELISA ... 20

4. Cytokine analysis secreted from splenocytes of the mouse vaccinated with V1-V3 ... 27

5. Flow cytometry ... 28

6. Experiments using humanized mice ... 29

6-1. Preparation of humanized mice ... 29

6-2. Immunization ... 29

6-3. ELISA ... 29

6-4. Splenocyte analysis ... 30

6-5. ELISpot ... 42

6-6. Cytokine analysis using LEGENDplex ... 44

7. References ... 46

1. Materials

1-1. Synthesis of Materials

Pam$_3$CSK$_4$–CH401 (**1**) and Pam–CH401 (**2**) were synthesized according to our previous report.[1] CH401-conjugated BSA was prepared according to the previous report.[1]

1-2. Protocol for the preparation of V1-V7

<Structure of used lipids>

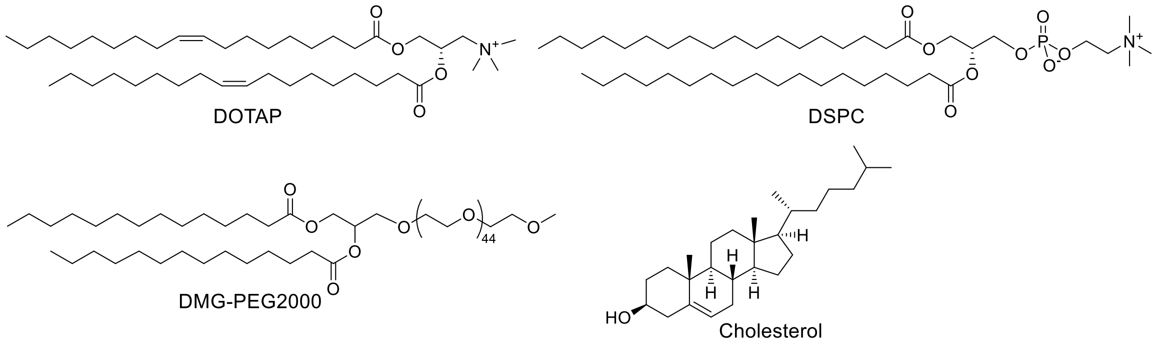

<Summary table of vaccine entries>

Generation	Vaccine entries	LNP formulation lipid composition DSPC/cholesterol/DMG-PEG2000	Antigen	Adjuvant
1st	**V1**	50/10/38.5/1.5	Pam–CH401	
	**V2**		Pam$_3$CSK$_4$–CH401	Pam$_3$CSK$_4$
	**V3**	35/25/38.5/1.5	Pam–CH401	
2nd	**V2’**	50/10/38.5/1.5	Pam$_3$CSK$_4$–CH401	
	**V4**		Pam$_3$CSK$_4$–CH401	α-GalCer
	**V5**		Pam$_3$CSK$_4$–CH401	MPLA
	**V6**		Pam$_3$CSK$_4$–CH401	CpG ODN 1826*
	**V7**		Pam$_3$CSK$_4$–CH401	MPLA, CpG ODN 1826*
Molecular ratio (mol%)		100	1	1

*CpG ODN 1826 amount: nitrogen/phosphate [N/P] ratio was adjusted to 6

<Preparation of **V1**>

The stock solution of DOTAP in DMSO (20 mM, 125 μL), the stock solution of DSPC in ethanol (20 mM, 25 μL), the stock solution of cholesterol in ethanol (20 mM, 96.25 μL), the stock solution of DMG-PEG(2000) in DMSO (5 mM, 15 μL), the stock solution of Pam–CH401 (**2**) in DMSO (0.4 mM, 125 μL), ethanol (378.5 μL), and DMSO (485 μL) were mixed to give a lipids solution. This lipid solution and 25 mM acetate buffer (pH 4.0) were introduced into the microfluidic iLiNP device[2] at a flow rate of 125 μL/min and 375 μL/min, respectively. These solutions were injected by micro syringes using syringe pumps (70-2208, Harvard Apparatus,

Massachusetts, US). The mixed solution from the outlet was collected in the tube and dialyzed against 500 mL of PBS at 4 ºC for 12 h using a dialysis membrane (042-30913, Size 8, molecular weight cutoff = 14 kDa, FUJIFILM Wako Pure Chemical Co., Ltd., Tokyo, Japan). This dialysis step was repeated once. The resultant solution was transferred to Amicon® Ultra-15 (UFC910024, molecular weight cutoff = 100 kDa) and centrifuged at 1,000×g at 4 °C. The concentration of Pam–CH401 (**2**) in lipid nanoparticle (LNP) solution was quantified by HPLC (column: COSMOSIL 5C4-AR-300 2.0×250 mm column (37959-01, Nacalai Tesque, Kyoto, Japan), solvent A: 0.1% TFA in water/acetonitrile/isopropanol (8/1/1), solvent B: 0.1% TFA in acetonitrile/isopropanol (1/1), eluent: linear gradient of 2% to 98% solvent B over 48 min, detection: 220 nm UV, device: Prominence UFLC (SHIMADZU, Kyoto, Japan)). The concentration of Pam–CH401 (**2**) in LNP was adjusted to 35 μM in PBS for immunization.

<Preparation of **V2**>

The stock solution of DOTAP in ethanol (20 mM, 200 μL), the stock solution of DSPC in ethanol (20 mM, 40 μL), the stock solution of cholesterol in ethanol (20 mM, 154 μL), the stock solution of DMG-PEG(2000) in ethanol (5 mM, 24 μL), the stock solution of Pam_3CSK_4–CH401 (**1**) in ethanol (0.4 mM, 200 μL), and ethanol (1382 μL) were mixed to give a lipid solution. This lipid solution and 25 mM acetate buffer (pH 4.0) were introduced into the microfluidic iLiNP device[2] at a flow rate of 125 μL/min and 375 μL/min, respectively. These solutions were injected by micro syringes using syringe pumps (70-2208, Harvard Apparatus, Massachusetts, US). The mixed solution from the outlet was collected in the tube and dialyzed against 500 mL of PBS at 4 ºC for 12 h using a dialysis membrane (042-30913, Size 8, molecular weight cutoff = 14 kDa, FUJIFILM Wako Pure Chemical Co., Ltd., Tokyo, Japan). This dialysis step was repeated once. 8 mL of the resultant solution was transferred to Amicon® Ultra-15 (UFC910024, molecular weight cutoff = 100 kDa) and centrifuged at 1,000×g at 4 °C. The concentration of Pam_3CSK_4–CH401 (**1**) in LNP solution was quantified by HPLC (column: COSMOSIL 5C4-AR-300 2.0×250 mm column (37959-01, Nacalai Tesque, Kyoto, Japan), flow rate: 0.5 mL/min, solvent A: 0.1% TFA in water/acetonitrile/isopropanol (8/1/1), solvent B: 0.1% TFA in acetonitrile/isopropanol (1/1), eluent: linear gradient of 60% to 98% solvent B over 19 min and then isocratic eluent of 98% solvent B in 11 min, detection: 220 nm UV, device: Prominence UFLC (SHIMADZU, Kyoto, Japan)). The concentration of Pam_3CSK_4–CH401 (**1**) was adjusted to 35 μM in PBS for immunization.

<Preparation of **V3**>

The stock solution of DOTAP in DMSO (20 mM, 37.5 μL), the stock solution of DSPC in ethanol (20 mM, 112.5 μL), the stock solution of cholesterol in ethanol (20 mM, 96.25 μL), the stock solution of DMG-PEG(2000) in DMSO (5 mM, 15 μL), the stock solution of Pam–CH401 (**2**) in DMSO (0.4 mM, 125 μL), the stock solution of Pam_3CSK_4 in DMSO (0.4 mM, 125 μL), ethanol (291.25 μL), and DMSO (447.5 μL) were mixed to give a lipid solution. This lipid solution and 25 mM acetate buffer (pH 4.0) were introduced into the microfluidic iLiNP device[2] at a flow rate of 125 μL/min and 375 μL/min, respectively. These solutions were injected by micro syringes using syringe pumps (70-2208, Harvard Apparatus, Massachusetts, US). The mixed solution from the outlet was collected in the tube and dialyzed against 500 mL of PBS at 4 ºC for 12 h using a

dialysis membrane (042-30913, Size 8, molecular weight cutoff = 14 kDa, FUJIFILM Wako Pure Chemical Co., Ltd., Tokyo, Japan). This dialysis step was repeated once. The resultant solution was transferred to Amicon® Ultra-15 (UFC910024, molecular weight cutoff = 100 kDa) and centrifuged at 1,000×g at 4 °C. The concentration of Pam–CH401 (**2**) in LNP solution was quantified by HPLC (column: COSMOSIL 5C4-AR-300 2.0×250 mm column (37959-01, Nacalai Tesque, Kyoto, Japan), solvent A: 0.1% TFA in water/acetonitrile/isopropanol (8/1/1), solvent B: 0.1% TFA in acetonitrile/isopropanol (1/1), eluent: linear gradient of 2% to 98% solvent B over 48 min, detection: 220 nm UV, device: Prominence UFLC (SHIMADZU, Kyoto, Japan)). The concentration of Pam–CH401 (**2**) was adjusted to 35 μM in PBS for immunization. Incorporation of Pam_3CSK_4 into LNP was confirmed by HPLC (column: COSMOSIL 5C4-AR-300 2.0×250 mm column (37959-01, Nacalai Tesque, Kyoto, Japan), solvent A: 0.1% TFA in water/acetonitrile/isopropanol (8/1/1), solvent B: 0.1% TFA in acetonitrile/isopropanol (1/1), eluent: linear gradient of 2% to 98% solvent B, detection: 220 nm UV, device: Prominence UFLC (SHIMADZU, Kyoto, Japan)).

<Preparation of **V2’**>

The stock solution of DOTAP in ethanol (20 mM, 20 μL), the stock solution of DSPC in ethanol (10 mM, 8 μL), the stock solution of cholesterol in ethanol (20 mM, 15.4 μL), the stock solution of DMG-PEG(2000) in ethanol (5 mM, 2.4 μL), the stock solution of Pam_3CSK_4–CH401 (**1**) in ethanol (0.4 mM, 20 μL), and ethanol (134.2 μL), were mixed to give a lipid solution. This lipid solution and 25 mM acetate buffer (pH 4.0) were introduced into the microfluidic iLiNP device[2] at a flow rate of 25 μL/min and 75 μL/min, respectively. These solutions were injected by micro syringes using Pump 11 Elite Syringe Pumps (70-4500, Harvard Apparatus, Massachusetts, US). The mixed solution from the outlet was collected in the tube and dialyzed against 500 mL of PBS at 4 ºC for 15 h using a dialysis membrane (042-30913, Size 8, molecular weight cutoff 14 kDa, FUJIFILM Wako Pure Chemical Co., Ltd., Tokyo, Japan). The concentration of Pam_3CSK_4–CH401 (**1**) in LNP solution was quantified by HPLC (column: COSMOSIL 5C4-AR-300 2.0×250 mm column (37959-01, Nacalai Tesque, Kyoto, Japan), flow rate: 0.5 mL/min, solvent A: 0.1% TFA in water/acetonitrile/isopropanol (8/1/1), solvent B: 0.1% TFA in acetonitrile/isopropanol (1/1), eluent: linear gradient of 60% to 98% solvent B over 19 min and then isocratic eluent of 98% solvent B in 11 min, detection: 220 nm UV, device: Prominence UFLC (SHIMADZU, Kyoto, Japan)). The concentration of Pam_3CSK_4–CH401 (**1**) was adjusted to 3.5 μM in PBS for immunization.

<Preparation of **V4**>

The stock solution of DOTAP in ethanol (20 mM, 20 μL), the stock solution of DSPC in ethanol (10 mM, 8 μL), the stock solution of cholesterol in ethanol (20 mM, 15.4 μL), the stock solution of DMG-PEG(2000) in ethanol (5 mM, 2.4 μL), the stock solution of α-GalCer in DMSO (0.4 mM, 20 μL), the stock solution of Pam_3CSK_4–CH401 (**1**) in ethanol (0.4 mM, 20 μL), and ethanol (114.2 μL), were mixed to give a lipid solution. This lipid solution and 25 mM acetate buffer (pH 4.0) were introduced into the microfluidic iLiNP device[2] at a flow rate of 25 μL/min and 75 μL/min, respectively. These solutions were injected by micro syringes using Pump 11 Elite Syringe Pumps (70-4500, Harvard Apparatus, Massachusetts, US). The mixed solution from the outlet was

collected in the tube and dialyzed against 500 mL of PBS at 4 ºC for 15 h using a dialysis membrane (042-30913, Size 8, molecular weight cutoff 14 kDa, FUJIFILM Wako Pure Chemical Co., Ltd., Tokyo, Japan). The concentration of Pam_3CSK_4–CH401 (**1**) in LNP solution was quantified by HPLC (column: COSMOSIL 5C4-AR-300 2.0×250 mm column (37959-01, Nacalai Tesque, Kyoto, Japan), flow rate: 0.5 mL/min, solvent A: 0.1% TFA in water/acetonitrile/isopropanol (8/1/1), solvent B: 0.1% TFA in acetonitrile/isopropanol (1/1), eluent: linear gradient of 60% to 98% solvent B over 19 min and then isocratic eluent of 98% in 11 min, detection: 220 nm UV, device: Prominence UFLC (SHIMADZU, Kyoto, Japan)). The concentration of α-GalCer in LNP solution was quantified by LC–MS (column: Inertsustain C18 3 μm non-metal PEEK 2.1×100 mm column (5020-87413, GL Sciences, Tokyo, Japan), flow rate: 0.2 mL/min, solvent A: 10 mM NH_3 in water/methanol (2/8), solvent B: 10 mM NH_3 in 2-propanol, eluent: isocratic eluent of 0 % solvent B in 1 min, linear gradient of 0% to 95% solvent B over 34 min, and then isocratic eluent of 95% solvent B in 5 min, detection: mass peak area, LC–MS device: micrOTOF QII (Bruker, Massachusetts, US) equipped with Agilent 1260 Infinity LC system (Agilent Technologies, California, US)). The concentration of Pam_3CSK_4–CH401 (**1**) was adjusted to 3.5 μM in PBS for immunization. The concentration of α-GalCer in LNP solution was quantified by LC-MS (1st immunization sample: 3.1 μM, 2nd immunization sample: 3.4 μM, 3rd immunization sample: 1.9 μM, column: InertSustain C18 3 μm non-metal PEEK 2.1×100 mm column (5020-87413, GL Sciences, Tokyo, Japan), flow rate: 0.2 mL/min, solvent A: 10 mM NH_3 in water/methanol (2/8), solvent B: 10 mM NH_3 in 2-propanol, eluent: isocratic eluent of 0 % solvent B in 1 min, linear gradient of 0% to 95% solvent B over 34 min, and then isocratic eluent of 95% solvent B in 5 min, detection: mass peak area, LC–MS device: micrOTOF QII (Bruker, Massachusetts, US) equipped with Agilent 1260 Infinity LC system (Agilent Technologies, California, US)).

<Preparation of **V5**>

The stock solution of DOTAP in ethanol (20 mM, 20 μL), the stock solution of DSPC in ethanol (10 mM, 8 μL), the stock solution of cholesterol in ethanol (20 mM, 15.4 μL), the stock solution of DMG-PEG(2000) in ethanol (5 mM, 2.4 μL), the stock solution of MPLA in DMSO (0.4 mM, 20 μL), the stock solution of Pam_3CSK_4–CH401 (**1**) in ethanol (0.4mM, 20 μL), and ethanol (114.2 μL) were mixed to give a lipid solution. This lipid solution and 25 mM acetate buffer (pH 4.0) were introduced into the microfluidic iLiNP device[2] at a flow rate of 25 μL/min and 75 μL/min, respectively. These solutions were injected by micro syringes using Pump 11 Elite Syringe Pumps (70-4500, Harvard Apparatus, Massachusetts, US). The mixed solution from the outlet was collected in the tube and dialyzed against 500 mL of PBS at 4 ºC for 15 h using a dialysis membrane (042-30913, Size 8, molecular weight cutoff 14 kDa, FUJIFILM Wako Pure Chemical Co., Ltd., Tokyo, Japan). The concentration of Pam_3CSK_4–CH401 (**1**) in LNP solution was quantified by HPLC (column: COSMOSIL 5C4-AR-300 2.0×250 mm column (37959-01, Nacalai Tesque, Kyoto, Japan), flow rate: 0.5 mL/min, solvent A: 0.1% TFA in water/acetonitrile/isopropanol (8/1/1), solvent B: 0.1% TFA in acetonitrile/isopropanol (1/1), eluent: linear gradient of 60% to 98% solvent B over 19 min and then isocratic eluent of 98% in 11 min, detection: 220 nm UV, device: Prominence UFLC (SHIMADZU, Kyoto, Japan)). The concentration of Pam_3CSK_4–CH401 (**1**) was adjusted to 3.5 μM in PBS for immunization. The concentration of MPLA in LNP

solution was quantified by LC–MS (1st immunization sample: 3.6 μM, 2nd immunization sample: 2.8 μM, 3rd immunization sample: 2.9 μM, column: Inertsustain C18 3 μm non-metal PEEK 2.1×100 mm column (5020-87413, GL Sciences, Tokyo, Japan), flow rate: 0.2 mL/min, solvent A: 10 mM NH_3 in water/methanol (2/8), solvent B: 10 mM NH_3 in 2-propanol, eluent: isocratic eluent of 0 % solvent B in 1 min, linear gradient of 0% to 95% solvent B over 34 min, and then isocratic eluent of 95% solvent B in 5 min, detection: mass peak area, LC–MS device: micrOTOF QII (Bruker, Massachusetts, US) equipped with Agilent 1260 Infinity LC system (Agilent Technologies, California, US)).

<Preparation of **V6**>

The stock solution of DOTAP in ethanol (20 mM, 20 μL), the stock solution of DSPC in ethanol (10 mM, 8 μL), the stock solution of cholesterol in ethanol (20 mM, 15.4 μL), the stock solution of DMG-PEG(2000) in ethanol (5 mM, 2.4 μL), the stock solution of Pam_3CSK_4–CH401 (**1**) in ethanol (0.4mM, 20 μL), and ethanol (134.2 μL), were mixed to give a lipid solution. This lipid solution and 25 mM acetate buffer (pH 4.0) containing 35.3 μg/mL CpG ODN 1826 were introduced into the microfluidic iLiNP device[2] at a flow rate of 25 μL/min and 75 μL/min, respectively. These solutions were injected by micro syringes using Pump 11 Elite Syringe Pumps (70-4500, Harvard Apparatus, Massachusetts, US). The mixed solution from the outlet was collected in the tube and dialyzed against 500 mL of PBS at 4 ºC for 15 h using a dialysis membrane (042-30913, Size 8, molecular weight cutoff 14 kDa, FUJIFILM Wako Pure Chemical Co., Ltd., Tokyo, Japan). The concentration of Pam_3CSK_4–CH401 (**1**) in LNP solution was quantified by HPLC (column: COSMOSIL 5C4-AR-300 2.0×250 mm column (37959-01, Nacalai Tesque, Kyoto, Japan), flow rate: 0.5 mL/min, solvent A: 0.1% TFA in water/acetonitrile/isopropanol (8/1/1), solvent B: 0.1% TFA in acetonitrile/isopropanol (1/1), eluent: linear gradient of 60% to 98% solvent B over 19 min and then isocratic eluent of 98% in 11 min, detection: 220 nm UV, device: Prominence UFLC (SHIMADZU, Kyoto, Japan)). The concentration of Pam_3CSK_4–CH401 (**1**) was adjusted to 3.5 μM in PBS for immunization. The concentration of CpG ODN 1826 was quantified by the Ribogreen assay.

<Preparation of **V7**>

The stock solution of DOTAP in ethanol (20 mM, 20 μL), the stock solution of DSPC in ethanol (10 mM, 8 μL), the stock solution of cholesterol in ethanol (20 mM, 15.4 μL), the stock solution of DMG-PEG(2000) in ethanol (5 mM, 2.4 μL), the stock solution of MPLA in DMSO (0.4 mM, 20 μL), the stock solution of Pam_3CSK_4–CH401 (**1**) in ethanol (0.4mM, 20 μL), and ethanol (114.2 μL), were mixed to give a lipid solution. This lipid solution and 35.3 μg/mL CpG ODN 1826 in 25 mM acetate buffer were introduced into the microfluidic iLiNP device[2] at a flow rate of 25 μL/min and 75 μL/min, respectively. These solutions were injected by micro syringes using Pump 11 Elite Syringe Pumps (70-4500, Harvard Apparatus, Massachusetts, US). The mixed solution from the outlet was collected in the tube and dialyzed against 500 mL of PBS at 4 ºC for 15 h using a dialysis membrane (042-30913, Size 8, molecular weight cutoff 14 kDa, FUJIFILM Wako Pure Chemical Co., Ltd., Tokyo, Japan). The concentration of Pam_3CSK_4–CH401 (**1**) in LNP solution was quantified by HPLC (column: COSMOSIL 5C4-AR-300 2.0×250 mm column (37959-01, Nacalai Tesque, Kyoto, Japan), flow rate:

0.5 mL/min, solvent A: 0.1% TFA in water/acetonitrile/isopropanol (8/1/1), solvent B: 0.1% TFA in acetonitrile/isopropanol (1/1), eluent: linear gradient of 60% to 98% solvent B over 19 min and then isocratic eluent of 98% in 11 min, detection: 220 nm UV, device: Prominence UFLC (SHIMADZU, Kyoto, Japan)). The concentration of Pam_3CSK_4–CH401 (**1**) was adjusted to 3.5 µM in PBS for immunization. The concentration of MPLA in LNP solution was quantified by LC–MS (1st immunization sample: 1.9 µM, 2nd immunization sample: 1.6 µM, 3rd immunization sample: 1.7 µM, column: Inertsustain C18 3 µm non-metal PEEK 2.1×100 mm column (5020-87413, GL Sciences, Tokyo, Japan), flow rate: 0.2 mL/min, solvent A: 10 mM NH_3 in water/methanol (2/8), solvent B: 10 mM NH_3 in 2-propanol, eluent: isocratic eluent of 0 % solvent B in 1 min, linear gradient of 0% to 95% solvent B over 34 min, and then isocratic eluent of 95% solvent B in 5 min, detection: mass peak area, LC–MS device: micrOTOF QII (Bruker, Massachusetts, US) equipped with Agilent 1260 Infinity LC system (Agilent Technologies, California, US)). The concentration of CpG ODN 1826 was quantified by the Ribogreen® assay.

1-3. Ribogreen assay

The encapsulation of CpG ODN 1826 was determined using the Ribogreen fluorescence assay. 100 µL/well of the diluted LNP solutions in 10 mM HEPES buffer were applied to a Nunc™ F96 MicroWell™ Black polystyrene plate (237105, Thermo Fisher Scientific, Massachusetts, US). Then, two types of 10 mM HEPES buffer containing Quanti-i™ RiboGreen™ RNA reagent (R11491, Invitrogen (Thermo Fisher Scientific)) in the presence /absence of 0.1 w/v % Triton X-100 were added. After incubation at room temperature for 5min, fluorescence was measured by BioTek Cytation 5 plate reader (Agilent Technologies Inc., California, US) with λ_{Ex} = 480 nm and λ_{Em} = 520 nm. CpG ODN 1826 encapsulation efficiency was calculated from the following equation. For the CpG ODN 1826 standard curve, 2 mg/mL of CpG ODN 1826 was diluted with 10 mM HEPES buffer (pH 7.4) to 500 ng/mL.

$$\text{encapsulation efficiency (\%)} = \frac{\text{CpG ODN 1826 concentration of Triton }(+) - \text{CpG ODN 1826 concentration of Triton }(-)}{\text{CpG ODN 1826 concentration of Triton }(+)} \times 100$$

Table S1. The concentration of CpG ODN 1826 in the LNP solution of **V6** and **V7**. The data show the concentration of CpG ODN 1826 in the LNP solution with 3.5 µM of Pam_3CSK_4–CH401 (**1**).

	1st sample	2nd sample	3rd sample
**V6**	6.8 µg/mL (98%)	8.1 µg/mL (93%)	7.8 µg/mL (95%)
**V7**	6.1 µg/mL (98%)	7.6 µg/mL (94%)	8.6 µg/mL (95%)

( ): encapsulation efficiency

1-4. DLS analysis

<Protocol>

DLS was measured at 25 °C using Zetasizer Ultra (Malvern Panalytical, Worcestershire, UK) with an incident He-Ne laser (633 nm) and ZEN2112-Low volume glass cuvette cell. Backscattered light was detected. The instrument settings were optimized automatically using the ZS XPLORER software (Malvern Panalytical Ltd., Malvern, UK).

a) Size/nm

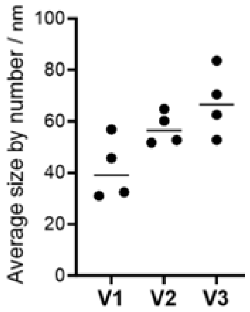

	V1	V2	V3
1st immunization	32	65	53
2nd immunization	31	52	84
3rd immunization	46	53	63
4th immunization	57	60	71

b) PI

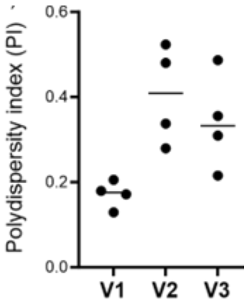

	V1	V2	V3
1st immunization	0.18	0.52	0.36
2nd immunization	0.21	0.34	0.49
3rd immunization	0.17	0.28	0.22
4th immunization	0.13	0.48	0.31

Figure S1. Summary of the DLS results of **V1-V3** in phosphate buffer (pH 7.4). Each point represents the result from five measurements. The data show four immunization samples. a) Average size by particle number. b) Polydispersity index (PI).

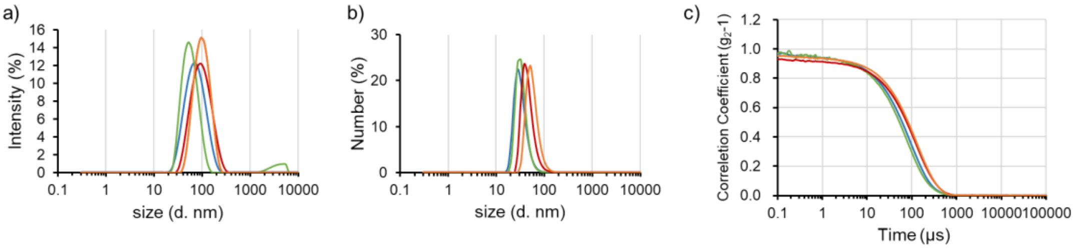

Figure S2. DLS results of **V1** in phosphate buffer (pH 7.4). Date represents the result from ten measurements. a) Size distribution by intensity. b) Size distribution by number. c) Auto Correlation Function. Blue line: 1st immunization sample, green line: 2nd immunization sample, red line: 3rd immunization sample, orange line: 4th immunization sample.

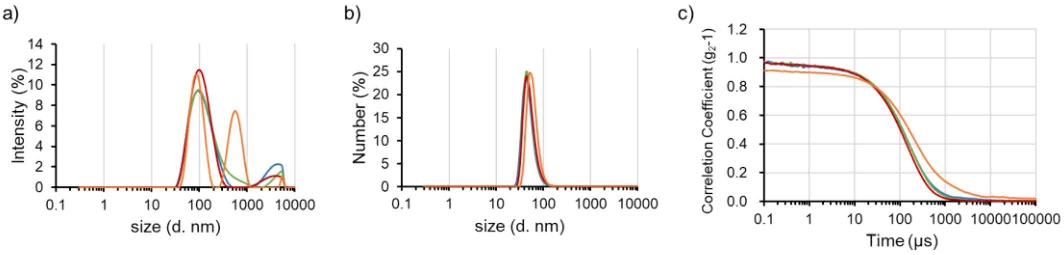

Figure S3. DLS results of **V2** in phosphate buffer (pH 7.4). Date represents the result from ten measurements. a) Size distribution by intensity. b) Size distribution by number. c) Auto Correlation Function. Blue line: 1st immunization sample, green line: 2nd immunization sample, red line: 3rd immunization sample, orange line: 4th immunization sample.

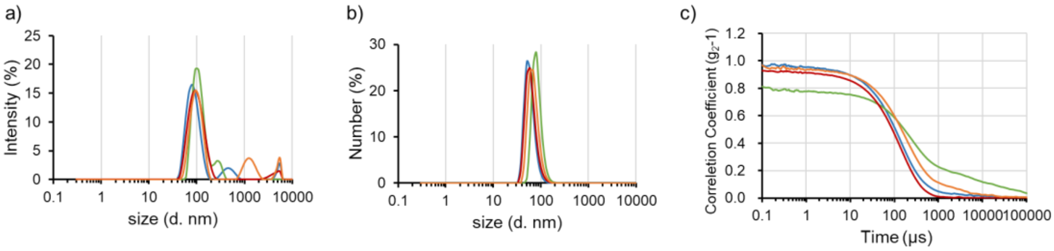

Figure S4. DLS results of **V3** in phosphate buffer (pH 7.4). Date represents the result from ten measurements. a) Size distribution by intensity. b) Size distribution by number. c) Auto Correlation Function. Blue line: 1st immunization sample, green line: 2nd immunization sample, red line: 3rd immunization sample, orange line: 4th immunization sample.

a) Size/nm

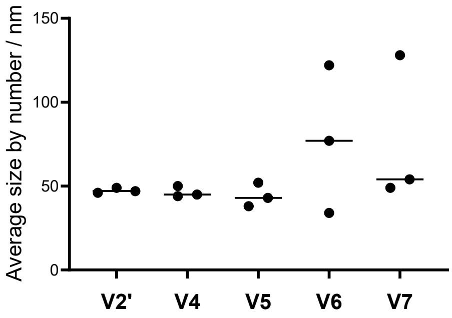

	V2'	V4	V5	V6	V7
1st immunization	47	44	43	34	54
2nd immunization	49	45	52	122	128
3rd immunization	46	50	38	77	49

b) PI

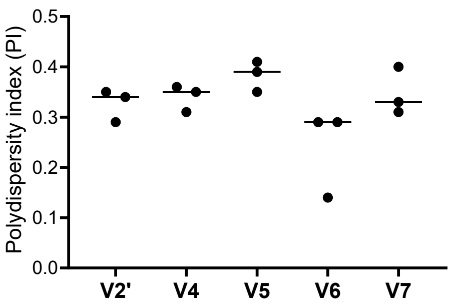

<PI>

	V2'	V4	V5	V6	V7
1st immunization	0.34	0.31	0.39	0.29	0.40
2nd immunization	0.29	0.35	0.35	0.29	0.33
3rd immunization	0.35	0.36	0.41	0.14	0.31

Figure S5. Summary of the DLS results of **V2'**, **V4-V7** in phosphate buffer (pH 7.4). Each point represents the result from five measurements. The data show three immunization samples. a) Average size by particle number. b) PI.

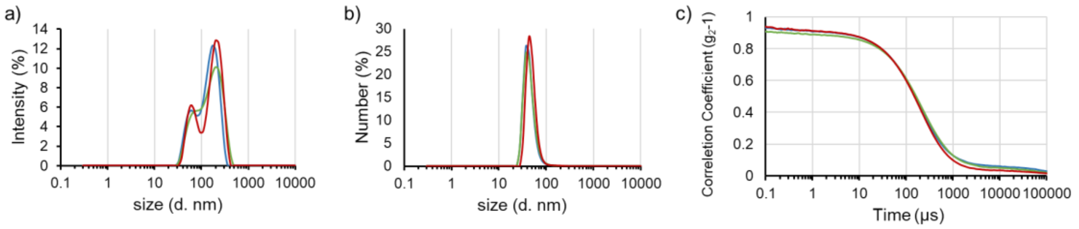

Figure S6. DLS results of **V4** in phosphate buffer (pH 7.4). a) Size distribution by intensity. b) Size distribution by number. c) Auto Correlation Function. Blue line: 1st immunization sample, green line: 2nd immunization sample, red line: 3rd immunization sample. Each result represents the average of five measurements.

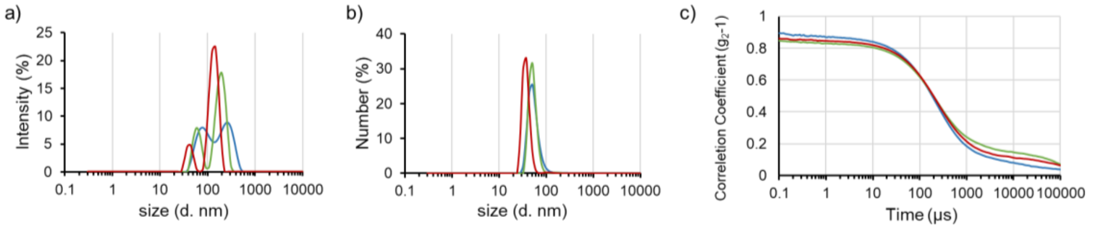

Figure S7. DLS results of **V5** in phosphate buffer (pH 7.4). a) Size distribution by intensity. b) Size distribution by number. c) Auto Correlation Function. Blue line: 1st immunization sample, green line: 2nd immunization sample, red line: 3rd immunization sample. Each result represents the average of five measurements.

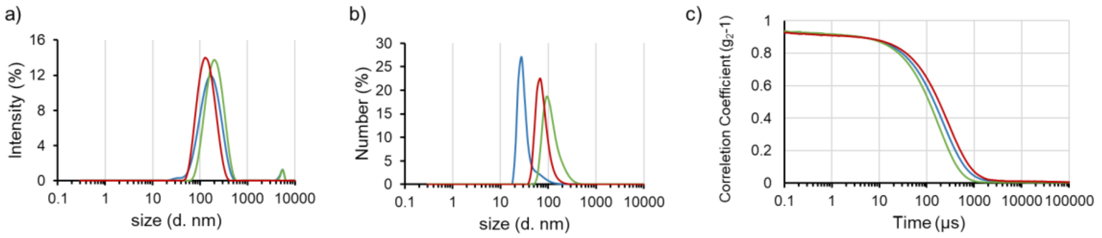

Figure S8. DLS results of **V6** in phosphate buffer (pH 7.4). a) Size distribution by intensity. b) Size distribution by number. c) Auto Correlation Function. Blue line: 1st immunization sample, green line: 2nd immunization sample, red line: 3rd immunization sample. Each result represents the average of five measurements.

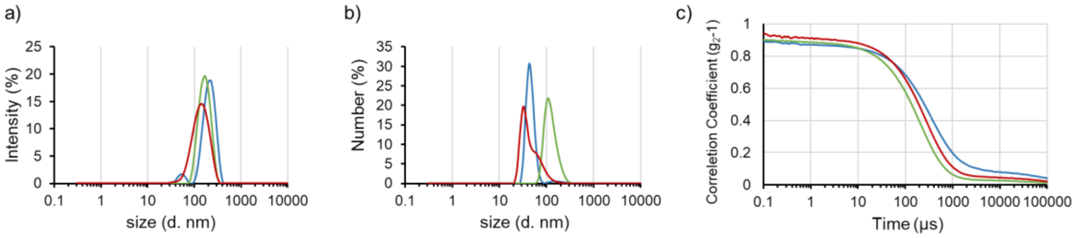

Figure S9. DLS results of **V7** in phosphate buffer (pH 7.4). a) Size distribution by intensity. b) Size distribution by number. c) Auto Correlation Function. Blue line: 1st immunization sample, green line: 2nd immunization sample, red line: 3rd immunization sample. Each result represents the average of five measurements.

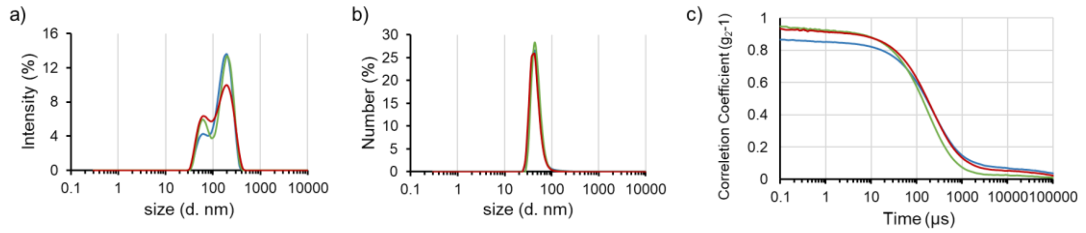

Figure S10. DLS results of **V2'** in phosphate buffer (pH 7.4). a) Size distribution by intensity. b) Size distribution by number. c) Auto Correlation Function. Blue line: 1st immunization sample, green line: 2nd immunization sample, red line: 3rd immunization sample. Each result represents the average of five measurements.

1-5. ζ-Potential

<Protocol>

Each vaccine sample in PBS (Pam3CSK4-CH401 or Pam-CH401 concentration: ~35 μM) was diluted 35-fold with 10 mM HEPES buffer (pH 7.4). The diluted samples were used for ζ-potential measurements. ζ-Potentials of each sample were measured at 25 °C using a Zetasizer Ultra (Malvern Panalytical, Worcestershire, UK) with a DTS1070 clear disposable zeta cell.

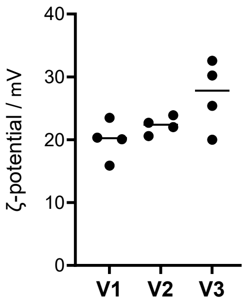

<ζ-potential/mV>

	V1	V2	V3
1st immunization	20.3	22.7	30.2
2nd immunization	15.9	23.9	32.6
3rd immunization	20.1	22.0	25.4
4th immunization	23.5	20.6	20.0

Figure S11. Summary of the ζ-potentials of **V1-V3**. The data show three immunization samples.

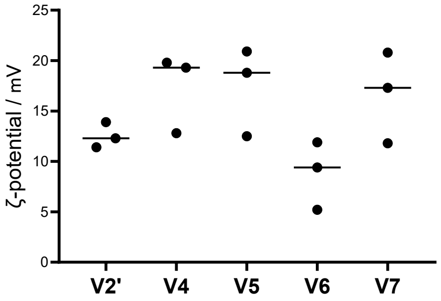

<ζ-potential/mV>

	V2'	V4	V5	V6	V7
1st immunization	12.3	19.3	12.5	5.2	17.3
2nd immunization	11.4	12.8	18.8	9.4	11.8
3rd immunization	13.9	19.8	20.9	11.9	20.8

Figure S12. ζ-potentials of **V2'**, **V4-V7**. The data show three immunization samples.

1-6. Nanoparticle stability

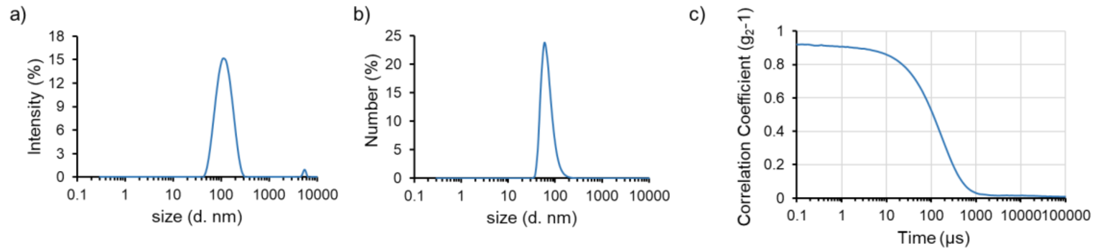

Figure S13. DLS results of **V1** after 8-day dialysis in phosphate buffer (pH 7.4) at 4 °C. Average size by particle number: 70 nm; PI: 0.26. a) Size distribution by intensity. b) Size distribution by number. c) Auto Correlation Function. Each result represents the average of five measurements.

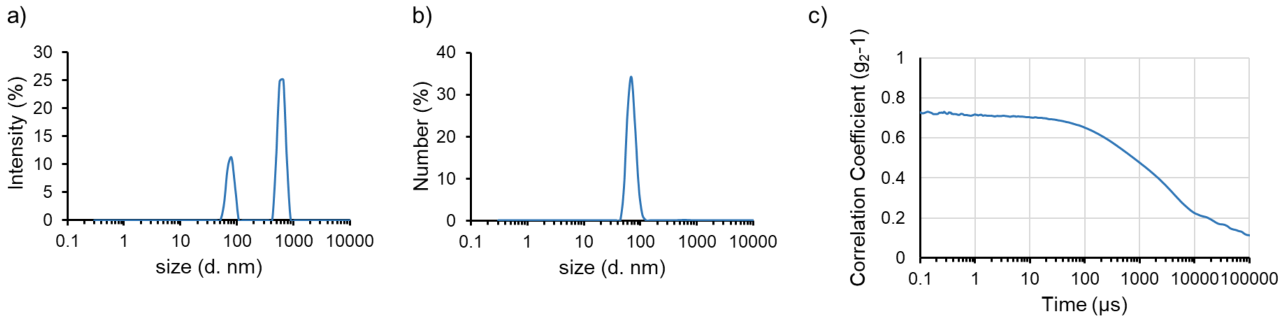

Figure S14. DLS results of **V2** after 8-day dialysis in phosphate buffer (pH 7.4) at 4 °C. Average size by particle number: 70 nm; PI: 0.91. a) Size distribution by intensity. b) Size distribution by number. c) Auto Correlation Function. Each result represents the average of five measurements.

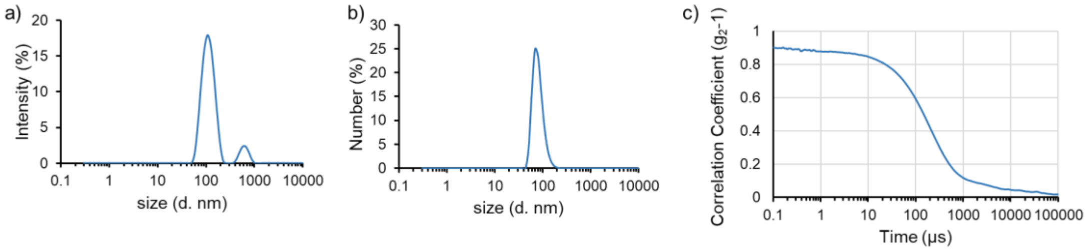

Figure S15. DLS results of **V3** after 8-day dialysis in phosphate buffer (pH 7.4) at 4 °C. Average size by particle number: 81 nm; PI: 0.38. a) Size distribution by intensity. b) Size distribution by number. c) Auto Correlation Function. Each result represents the average of five measurements.

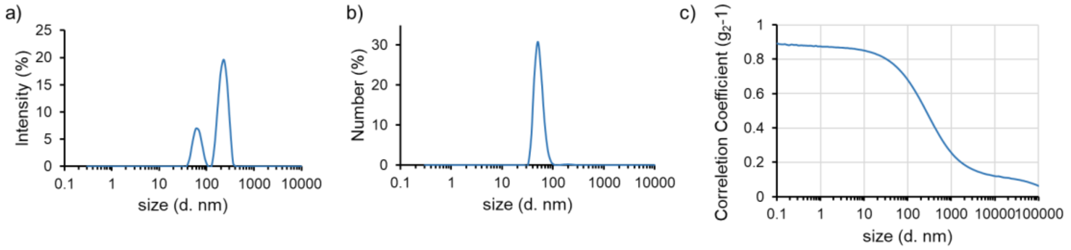

Figure S16. DLS results of **V4** in phosphate buffer (pH 7.4) after 1 month of storage at 4 °C (3rd immunization sample). Average size by particle number: 53 nm; PI: 0.40. a) Size distribution by intensity. b) Size distribution by number. c) Auto Correlation Function. Each result represents the average of five measurements.

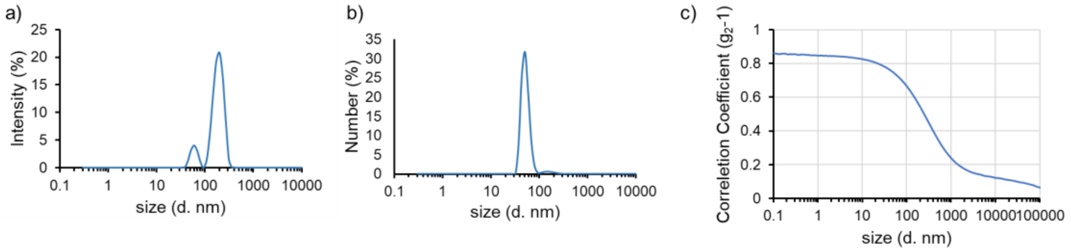

Figure S17. DLS results of **V5** in phosphate buffer (pH 7.4) after 1 month of storage at 4 °C (3rd immunization sample). Average size by particle number: 52 nm; PI: 0.38. a) Size distribution by intensity. b) Size distribution by number. c) Auto Correlation Function. Each result represents the average of five measurements.

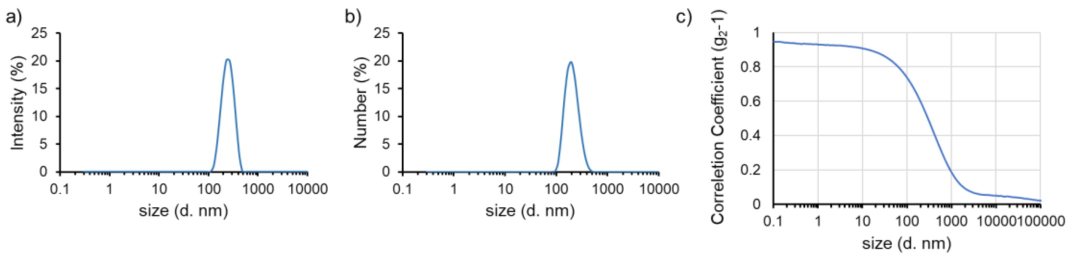

Figure S18. DLS results of **V6** in phosphate buffer (pH 7.4) after 1 month of storage at 4 °C (3rd immunization sample). Average size by particle number: 209 nm; PI: 0.40. a) Size distribution by intensity. b) Size distribution by number. c) Auto Correlation Function. Each result represents the average of five measurements.

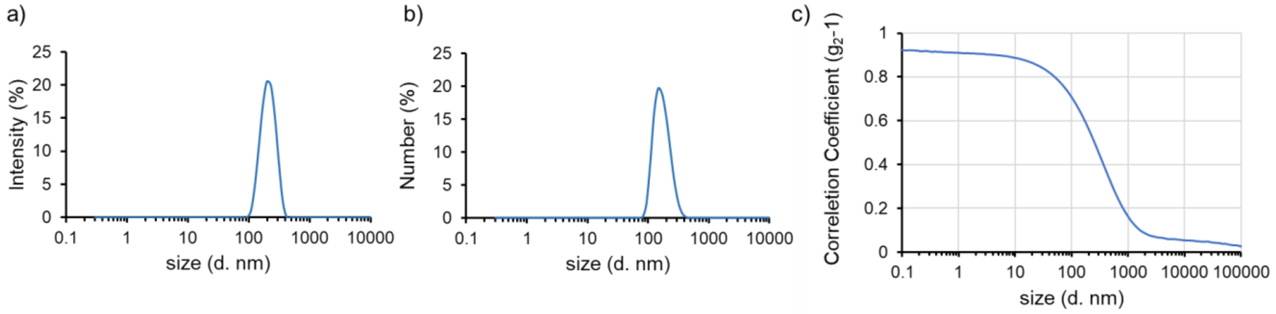

Figure S19. DLS results of **V7** in phosphate buffer (pH 7.4) after 1 month of storage at 4 °C (3rd immunization sample). Average size by particle number: 174 nm; PI: 0.35. a) Size distribution by intensity. b) Size distribution by number. c) Auto Correlation Function. Each result represents the average of five measurements.

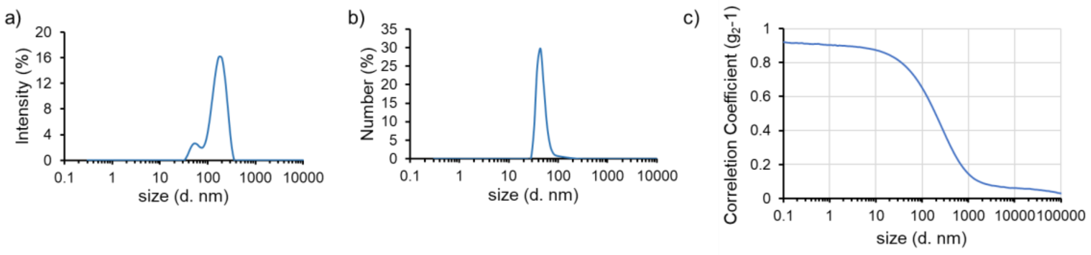

Figure S20. DLS results of **V2'** in phosphate buffer (pH 7.4) after 1 month of storage at 4 °C (3rd immunization sample). Average size by particle number: 48 nm; PI: 0.34. a) Size distribution by intensity. b) Size distribution by number. c) Auto Correlation Function. Each result represents the average of five measurements.

1-7. TEM analysis

<Protocol>

V1-V3

TEM imaging of **V1-V3** was commissioned to Hanaichi UltraStructure Research Institute, Co., Ltd. (Aichi,

Japan). The protocol is as follows. The droplet of the sample in PBS was placed on carbon-film grids. After incubation for 30 seconds, excess liquid was blotted off by touching one end of the grid with the filter paper. After the grid was partially dried, a drop of the staining solution (2 % phosphotungstic acid in water) was added to the grid and allowed for 10 seconds at 80 °C. After blotting off excess staining solution and drying the grid at rt, they were observed by Hitachi H-7600 electron microscopy (Hitachi Ltd., Tokyo, Japan) at an accelerating voltage of 100 kV.

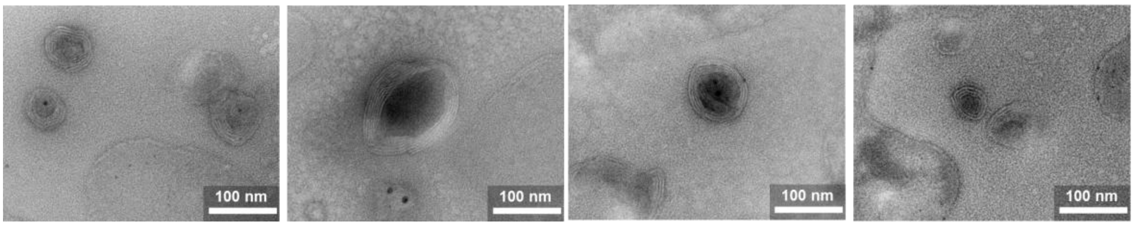

Figure S21. TEM Images of **V1**.

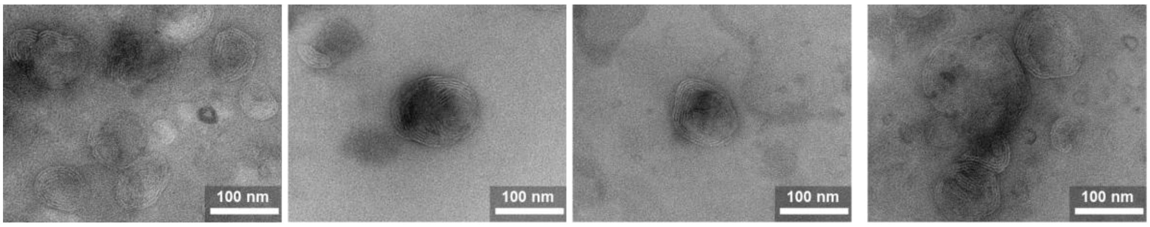

Figure S22. TEM Images of **V2**.

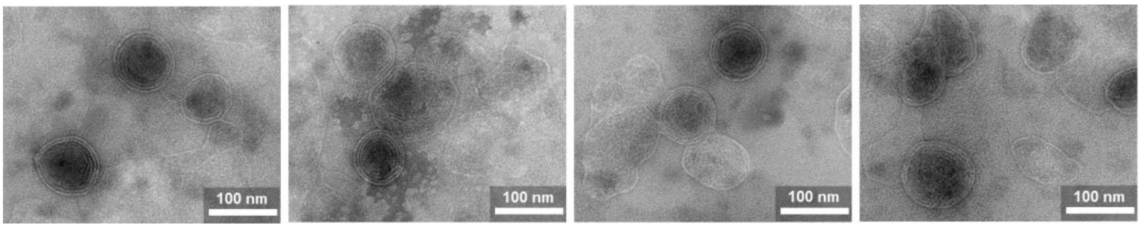

Figure S23. TEM Images of **V3**.

V2', **V4**-**V7**:

The 200-mesh micro copper grid (645, Nisshin EM Co., Ltd) was treated with soft plasma etching by SEDE-P (MEIWAFOSIS Co., Ltd., Tokyo, Japan) just before use. One μL of sample droplet at 3.5 μM of Pam$_3$CSK$_4$–CH401 (**1**) was applied on the grid. After incubation for a minute, excess liquid was blotted off by touching one end of the grid with the filter paper. After the grid was partially dried, 1 μL of five-fold diluted EM stainer (Nisshin EM Co., Ltd) in distilled water was applied and allowed for 20 seconds at 80 °C. After blotting off excess staining solution and drying the grid at rt, they were observed by JEM-2100 electron microscopy (JEOL, Tokyo, Japan) at an accelerating voltage of 200 kV.

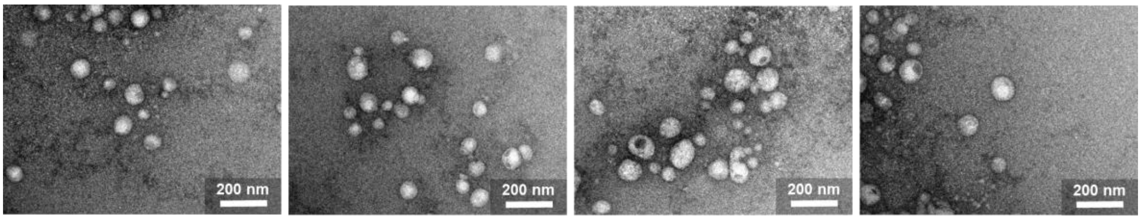

Figure S24. TEM Images of **V4**.

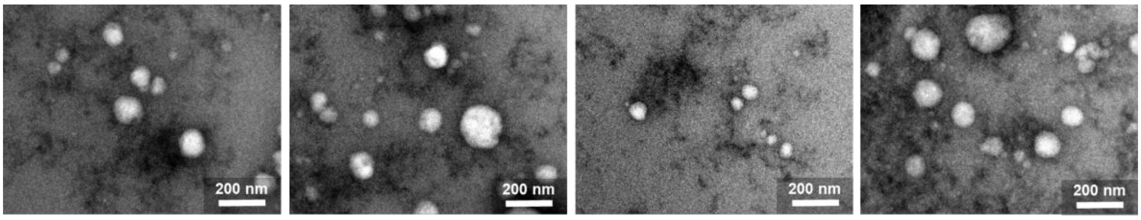

Figure S25. TEM Images of **V5**.

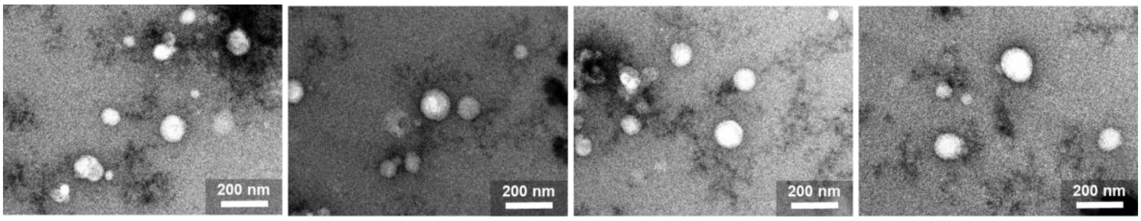

Figure S26. TEM Images of **V6**.

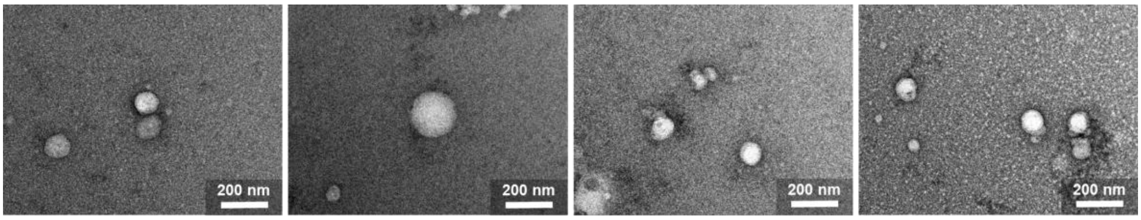

Figure S27. TEM Images of **V7**.

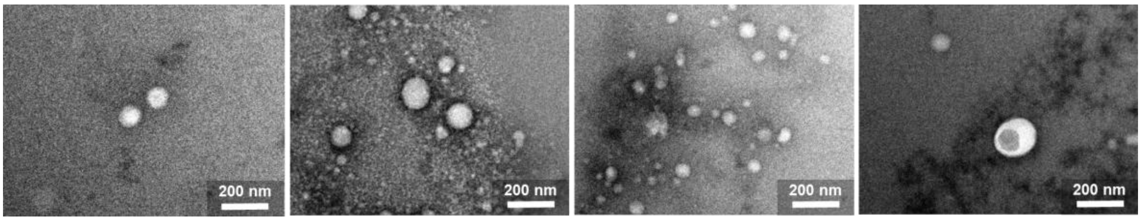

Figure S28. TEM Images of **V2’**.

2. Mice immunization

V1-V3:

All vaccines were administered within one week after preparation. BALB/c mice were obtained from Tokai University, School of Medicine. Experiments using BALB/c mice were approved in compliance with the Guidelines for the Care and Use of Laboratory Animals, and all animal studies were approved by the committees of the Tokai University, School of Medicine. The number of approvals is #235018. Each group of five wild-type BALB/c mice (8 weeks of age) was inoculated with an intraperitoneally (i.p.) injection of **V1-V3** on Day 0. The immunization schedule included boosting each mouse three times on Days 14, 28, and 42, respectively. Blood was collected from each mouse before immunization (blank controls) and one week after each immunization and was clotted to obtain plasmas that were stored at -30 °C before use.

We carefully checked the condition of the mice every day to avoid unexpected, intense immune responses in the mice. During immunization steps, partial toxicity was observed in mice administered with **V1** and **V2**, which is likely attributable to the nature of the cationic LNPs.[3] The details are as follows:

V1-immunized mice:

One mouse died after the third immunization on Day 38. The remaining four mice administered with **V1** showed no apparent adverse effects and remained in good health until Day 49.

V2-immunized mice:

One out of five mice weakened after the fourth immunization. Since the full vaccination schedule had been completed, all **V2**-treated mice were euthanized on Day 43.

V2', **V4-V7**:

All vaccines were administered within one week after preparation, during which stability was confirmed. Experiments using BALB/c mice were approved in compliance with the Guidelines for the Care and Use of Laboratory Animals, and all animal studies were approved by the committees of Japan SLC Inc. Biotechnical Center. The number of approvals is #BT23068. Each group of five wild-type BALB/c mice (8 weeks of age) was inoculated with an intraperitoneally (i.p.) injection on Day 0. The immunization schedule included boosting each mouse twice on Days 14, 28, respectively. Blood was collected from each mouse before immunization (blank controls) and on Days 7, 21, and 35, and was clotted to obtain plasmas that were stored at -30 °C before use.

3. ELISA

<Protocol>

V1-V3:

Corning® 96-well ELISA plates (polystyrene, Cat #9018, Corning Inc., NY, US) were coated with a solution of the BSA-CH401[1] (2 μg /mL, 50 μL per well) in the coating buffer (50 mM carbonate, pH 9.5) at 4 ºC for 16 h. Nonspecific sites were blocked with 1% (w/v) BSA (9018A3733-50G, Sigma Aldrich, St. Louis, US) in coating buffer at 37 ºC for 2 h, then washed three times with PBS at pH 7.4 using MW-96EX microplate washer (BIOTEC Co., Ltd., Tokyo, Japan). Subsequently, an individual mouse plasma with serial half-log dilutions from 1:50 to 1:32768400 in PBS containing 1% BSA was added to the coated plates (50 μL per well). The plates were incubated at 25 ºC for 2 h and then washed three times with PBS containing 0.05 % Tween-20 (PBST) using MW-96EX microplate washer. It was incubated at room temperature for 1 h with a 1:1000 diluted solution of HRP-linked goat anti-mouse IgG (A4416, Sigma-Aldrich), IgM (ab97230, abcam, Cambridge, UK) antibody, IgG1 (ab97240, abcam), IgG2a (ab97245, abcam), IgG2b (ab97250, abcam), or IgG3 (ab97260, abcam) antibody (50 μL per well), respectively. After the plates were washed five times with 0.05% PBST using MW-96EX microplate washer, 0.4 mg/mL *o*-phenylenediamine dihydrochloride (158-02151, OPD.2HCl, 5 mg/Tablet, FUJIFILM Wako Pure Chemical Corp., Osaka, Japan) in 0.05 M phosphate-citrate buffer at pH 5.0, with 0.4 μL/mL 30% H_2O_2 was added to the plates (100 μL per well) for 25 min at room temperature, 2.5 M aqueous sulfuric acid (50 μL per well) was subsequently added to stop the colorimetric reaction. Optical density (OD) was measured at 492 nm on BioTek Cytation 5 plate reader (Agilent Technologies Inc., California, US). For titer analysis, the OD values were plotted against the serum dilution numbers to obtain a best-fit logarithmic line. The equation of this line was used to calculate the dilution number at which an OD value of 0.1 for IgG, 0.2 for IgG subclasses, and 0.3 for IgM was achieved, and this dilution number was defined as the antibody titer.

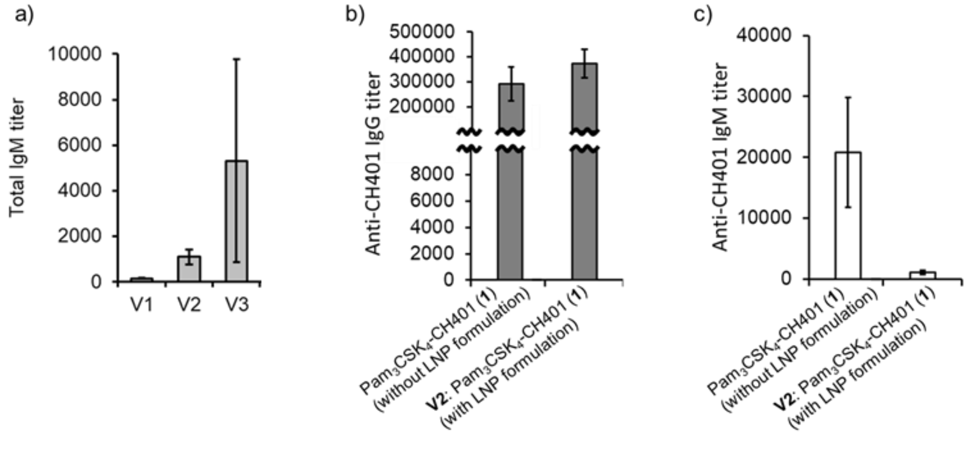

Figure S29. a) Anti-CH401 IgM titers of the plasma vaccinated with **V1-V3**. **V3**: n=5, **V1**, **V2**: n=4. b) Comparison of anti-IgG titers of the plasma immunized with non-LNP-formulated Pam_3CSK_4–CH401 (**1**) [1] and LNP-formulated Pam_3CSK_4–CH401 (**1**). c) Comparison of anti-IgG titers of the plasma immunized with non-LNP-formulated Pam_3CSK_4–CH401 (**1**) [1] and LNP-formulated Pam_3CSK_4–CH401 (**1**). The error bar represented the standard error of the mean.

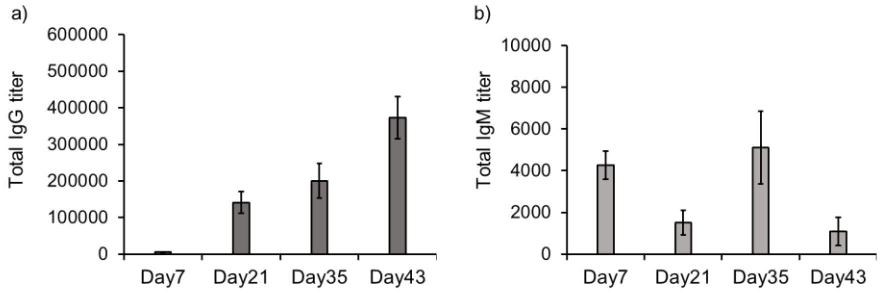

Figure S30. The booster effect of the anti-CH401 antibody titer of **V2**-immunized mice on Days 7, 21, 35, and 43. a) IgG and b) IgM. The data represent the results from five experiments (n = 5) for Days 7, 21, 35 and four experiments (n = 4) for Day 43. The error bar represented the standard error of the mean.

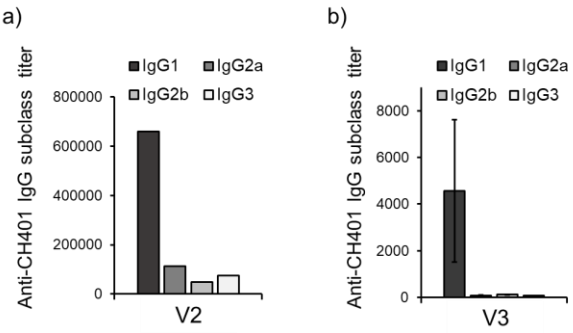

Figure S31. IgG subclass titers of plasma immunized with a) **V2** (n = 4), b) **V3** (n = 5) on Day 43 (**V2**)/Day 49 (**V3**). The error bar represented the standard error of the mean.

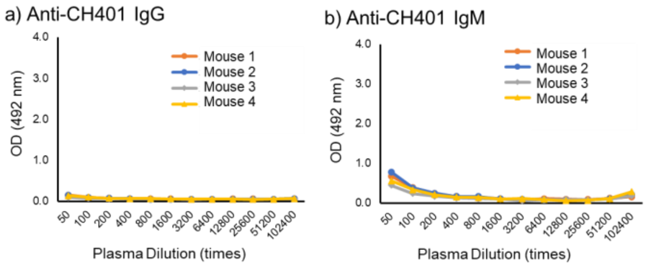

Figure S32. IgG and IgM antibody titers of plasma immunized with **V1** on Day 49 (n = 4). a) Anti-CH401 IgG, b) anti-CH401 IgM.

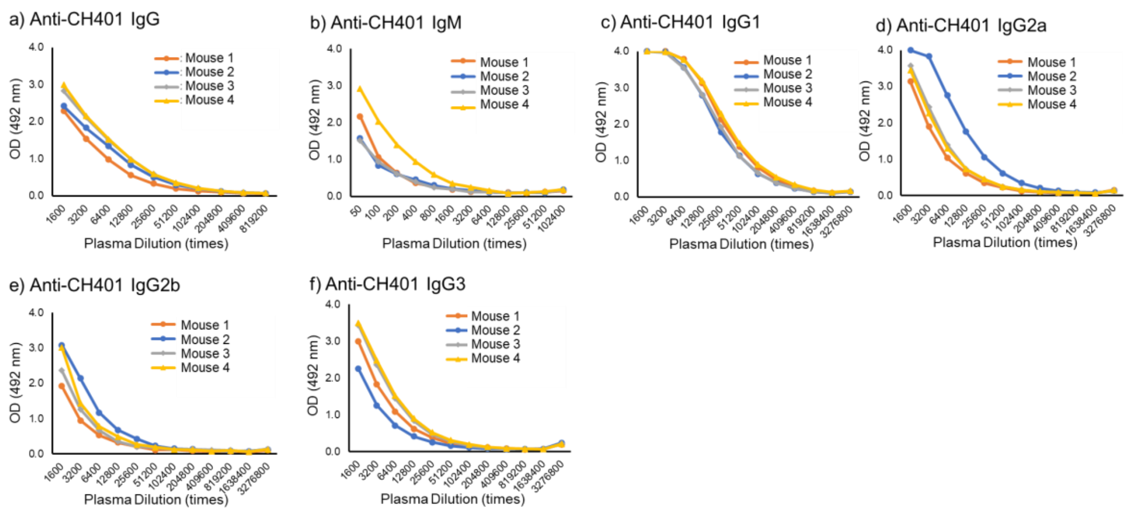

Figure S33. IgG and IgM antibody titers of plasma immunized with **V2** on Day 43 ($n = 4$). a) Anti-CH401 IgG, b) anti-CH401 IgM, c) anti-CH401 IgG1, d) anti-CH401 IgG2a, e) anti-CH401 IgG2b, f) anti-CH401 IgG3.

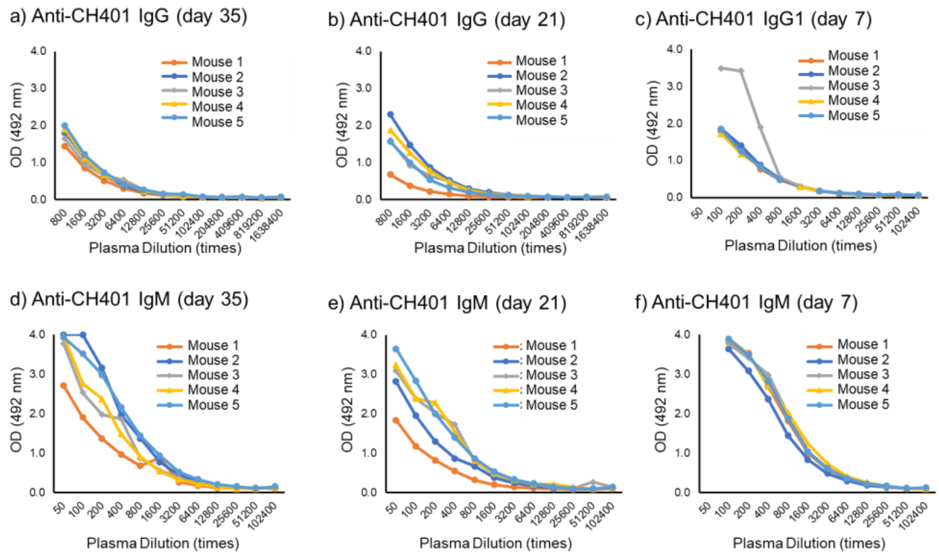

Figure S34. Anti-CH401 IgG and IgM booster effect of **V2** immunized mice on a, d) Day 35, b, e), Day 21 c, f), Day7 ($n = 5$).

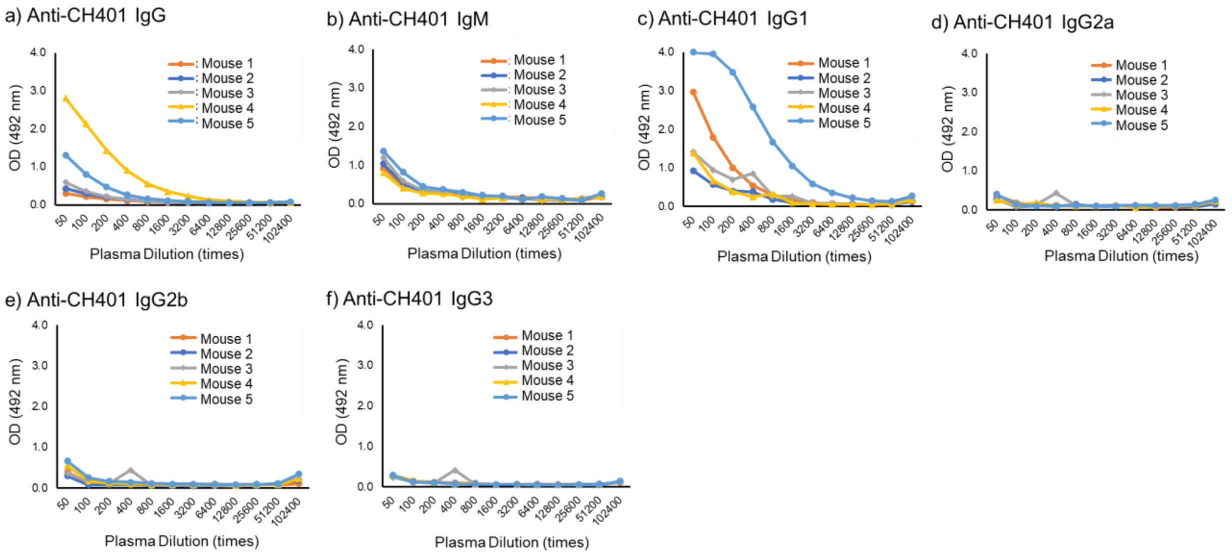

Figure S35. IgG and IgM antibody titers of plasma immunized with **V3** on Day 49 ($n = 5$). a) Anti-CH401 IgG, b) anti-CH401 IgM, c) anti-CH401 IgG1, d) anti-CH401 IgG2a, e) anti-CH401 IgG2b, f) anti-CH401 IgG3.

V2', V4-V7:

Corning® 96-well ELISA plates (polystyrene, Cat #9018, Corning Inc., NY, US) were coated with a solution of the BSA-CH401 (2 μg /mL, 50 μL per well) in the coating buffer (50 mM carbonate, pH 9.5) at 4 ºC for 16 h. Nonspecific sites were blocked with 1% (w/v) BSA (9018A3733-50G, Sigma Aldrich, St. Louis, US) in coating buffer at 37 ºC for 2 h, then washed three times with PBS at pH 7.4 using MW-96EX microplate washer (BIOTEC Co., Ltd., Tokyo, Japan). Subsequently, an individual mouse plasma with serial half-log dilutions from 1:100 to 1:819200 in PBS containing 1% BSA was added to the coated plates (50 μL per well). The plates were incubated at 25 ºC for 2 h and then washed three times with PBS containing 0.05 % Tween-20 (PBST) using MW-96EX microplate washer. It was incubated at room temperature for 1 h with a 1:1000 diluted solution of HRP-linked goat anti-mouse IgG (A4416, Sigma-Aldrich), IgM (ab97230, abcam, Cambridge, UK) antibody, IgG1 (ab97240, abcam), IgG2a (ab97245, abcam), IgG2b (ab97250, abcam), or IgG3 (ab97260, abcam) antibody (50 μL per well), respectively. After the plates were washed five times with 0.05% PBST using MW-96EX microplate washer, 0.4 mg/mL *o*-phenylenediamine dihydrochloride (158-02151, OPD.2HCl, 5 mg/Tablet, FUJIFILM Wako Pure Chemical Corp., Osaka, Japan) in 0.05 M phosphate-citrate buffer at pH 5.0, with 0.4 μL/mL 30% H_2O_2 was added to the plates (100 μL per well) for 25 min at room temperature, 2.5 M aqueous sulfuric acid (50 μL per well) was subsequently added to stop the colorimetric reaction. Optical density (OD) was measured at 492 nm on BioTek Cytation 5 plate reader (Agilent Technologies Inc., California, US). For titer analysis, the OD values were plotted against the serum dilution numbers to obtain a best-fit logarithmic line. The equation of this line was used to calculate the dilution number at which an OD value of 0.1 for IgG, 0.2 for IgG subclasses, and 0.3 for IgM was achieved, and this dilution number was defined as the antibody titer.

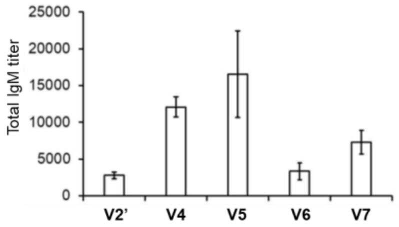

Figure S36. Anti-CH401 IgM titers of the plasma vaccinated with **V2'**, **V4-V3**. n=5. The error bar represented the standard error of the mean.

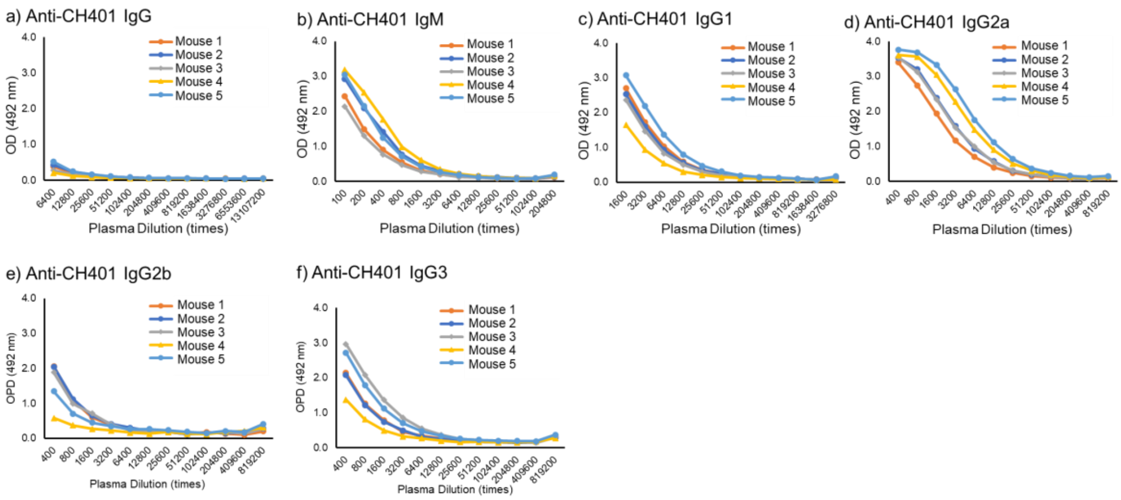

Figure S37. IgG and IgM antibody titers of plasma immunized with **V2'** on Day 35 (n = 5). a) Anti-CH401 IgG, b) anti-CH401 IgM, c) anti-CH401 IgG1, d) anti-CH401 IgG2a, e) anti-CH401 IgG2b, f) anti-CH401 IgG3.

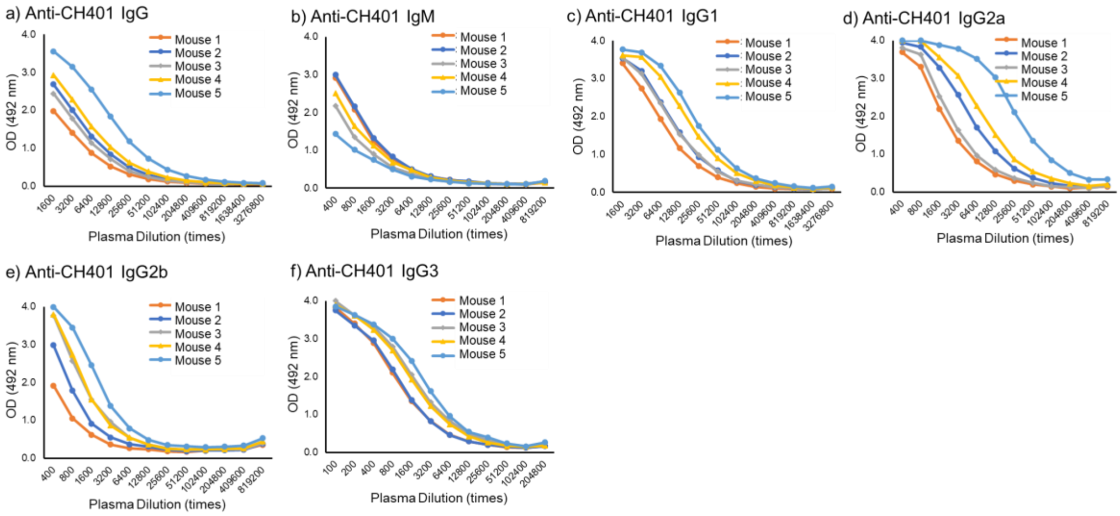

Figure S38. IgG and IgM antibody titers of plasma immunized with **V4** on Day 35 (n = 5). a) Anti-CH401 IgG, b) anti-CH401 IgM, c) anti-CH401 IgG1, d) anti-CH401 IgG2a, e) anti-CH401 IgG2b, f) anti-CH401 IgG3.

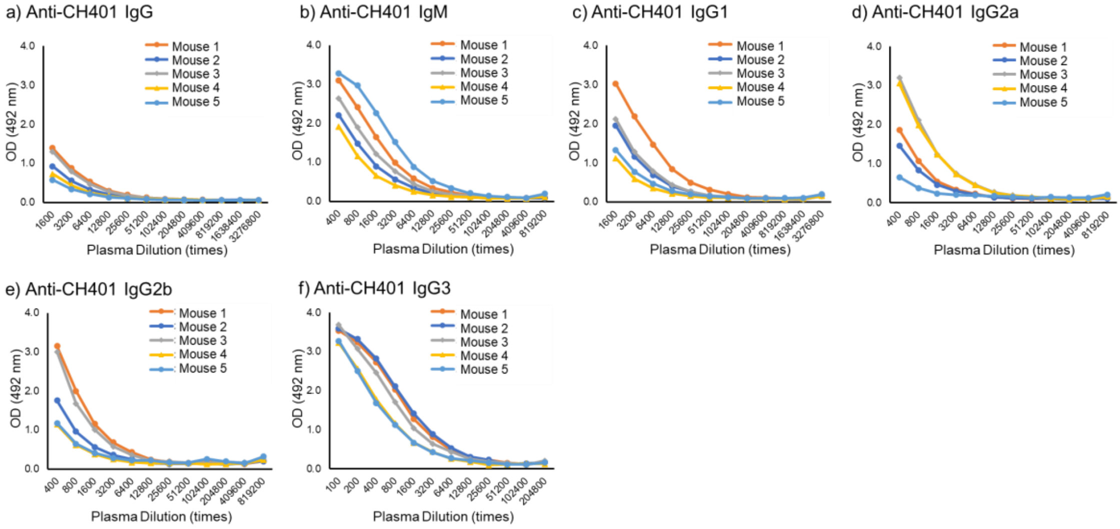

Figure S39. IgG and IgM antibody titers of plasma immunized with **V5** on Day 35 (n = 5). a) Anti-CH401 IgG, b) anti-CH401 IgM, c) anti-CH401 IgG1, d) anti-CH401 IgG2a, e) anti-CH401 IgG2b, f) anti-CH401 IgG3.

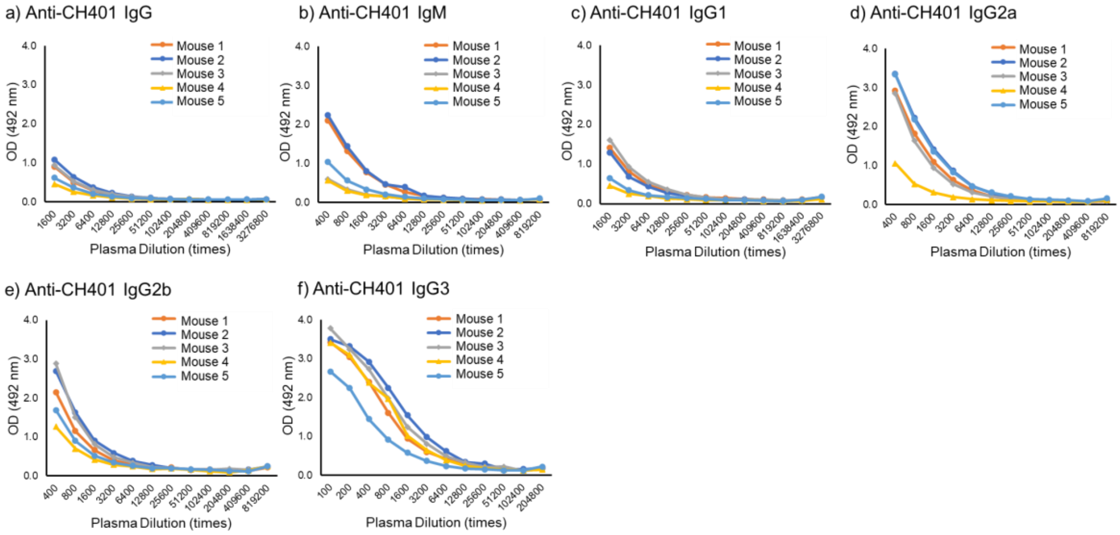

Figure S40. IgG and IgM antibody titers of plasma immunized with **V6** on Day 35 (n = 5). a) Anti-CH401 IgG, b) anti-CH401 IgM, c) anti-CH401 IgG1, d) anti-CH401 IgG2a, e) anti-CH401 IgG2b, f) anti-CH401 IgG3.

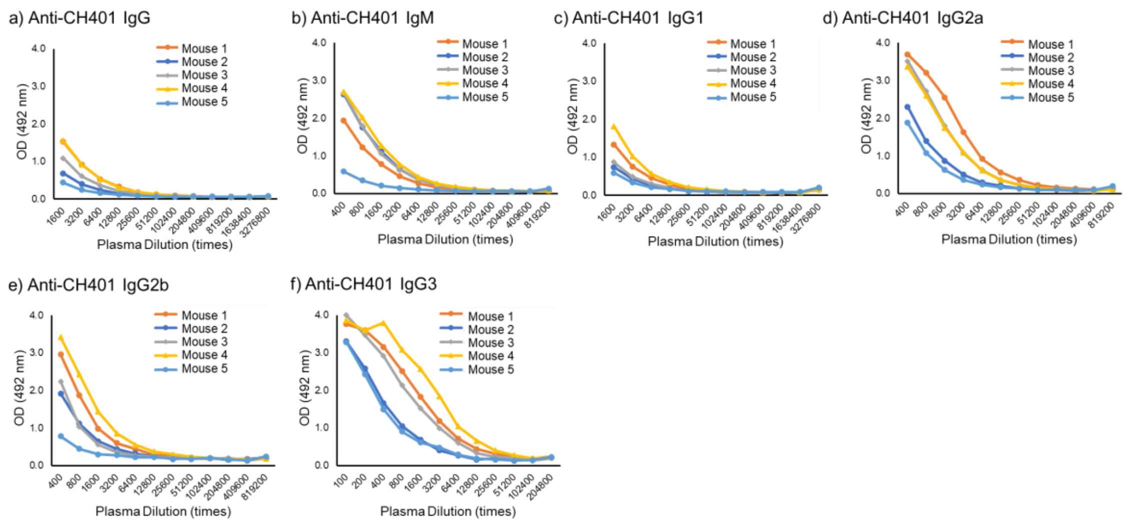

Figure S41. IgG and IgM antibody titers of plasma immunized with **V7** on Day 35 (*n* = 5). a) Anti-CH401 IgG, b) anti-CH401 IgM, c) anti-CH401 IgG1, d) anti-CH401 IgG2a, e) anti-CH401 IgG2b, f) anti-CH401 IgG3.

4. Cytokine analysis secreted from splenocytes of the mouse vaccinated with V1-V3

<Protocol>

The spleen cells of each mouse were collected on Day 43 (**V2**)/Day 49 (**V1** and **V3**), filtered with a mesh (77 μm), and centrifuged (800×g, 5 min). The precipitate was suspended with RBC Lysis Buffer (10 mL) and centrifuged (800×g, 5 min). The spleen cells were obtained as a precipitate, suspended in CELLBANKER, and stored in liquid N_2 before use.

The collected splenocytes stored in liquid N_2 were thawed and washed with RPMI-1640 containing 10% (v/v) FBS. 2.4×10^5 cells of each splenocyte were cultured with 1 μg/mL toxic shock syndrome toxin-1 (TSST-1, Toxin Technology Inc., Florida, USA) in RPMI-1640 containing 10% (v/v) FBS (120 μL per well) on IWAKI 48 well microplates (3830-048, AGC TECHNO GLASS Co., Ltd., Shizuoka, Japan). After incubation for 72 h at 37 °C, the supernatants were collected, and the cytokines in the supernatants were evaluated by LEGENDplex™ MU Th1/Th2 Panel (8-plex) w/VbPV03 (741054, BioLegend, California, US) according to the manufacturer's instructions. The panel included IFN-γ, TNF-α, IL-2, IL-4, IL-5, IL-6, IL-10, and IL-13. The data were acquired using BD FACSVerse™ (BD Biosciences). The acquired data were analyzed using LEGENDplex™ software (BioLegend).

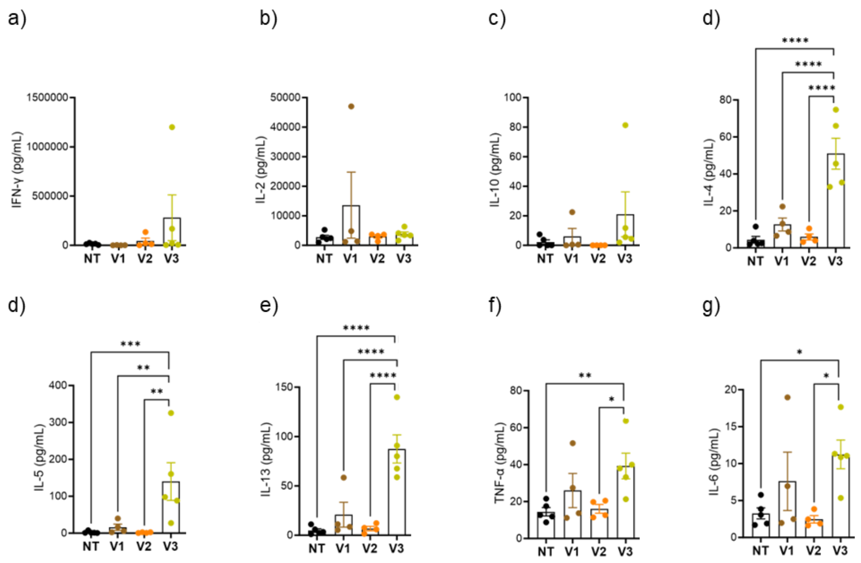

Figure S42. Cytokine profiles secreted by splenocytes collected from immunized mice. a) IFN-γ, b) IL-2, c) IL-10, d) IL-4, e) IL-5, f) IL-13, g) TNF-α, and h) IL-6. Each splenocyte was activated by TSST-1 for 72 h *ex vivo*. Each dot represented the result from **V1-V3**. **V3**: n=5, **V1**, **V2**: n=4. The bar chart represented the average of the dots. The error bar represented the standard error of the mean. Significance was determined via one-way ANOVA followed by Tukey's multiple comparison test with a single pooled variance.

5. Flow cytometry

<Protocol>

BT-474 cells (HER2 high expression) were cultured using RPMI containing 10% (v/v) FBS and 1% (v/v) penicillin-streptomycin. The cells were harvested by treating with trypsin-EDTA solution and transferred to 1.5 mL tubes at a density of 5.5×10^5 cells/mL in PBS containing 1% (w/v) BSA (FACS buffer). The cell number was counted by the EVE cell automatic cell counter (NanoEntek, Seoul, Korea). After the suction of FACS buffer, the cells were incubated with 100 μL of plasma collected from mice (dilution: 1:50; mixed plasma from a group (n = 5) was used) in FACS buffer. After being incubated on ice for 1 hour, the cells were washed three times with PBS. The resultant cells were incubated with 95 μL of Alexa Fluro®488-goat anti-mouse IgG (H+L) antibodies (1 μg/mL, 28175, Thermo Fisher Scientific) in FACS buffer on ice for 1 hour. The cells were washed three times with FACS buffer and suspended in 500 μL of FACS buffer. Ten thousand cells were measured for every flow cytometry analysis. Flow cytometry analysis was performed by Thermo Fisher Scientific Attune NxT Acoustic Focusing Cytometer.

6. Experiments using humanized mice

6-1. Preparation of humanized mice

This study was conducted by the guidelines of the Declaration of Helsinki and Japanese federal regulations required for the protection of human subjects. The study protocol was approved by Tokai University (12R-002/20R211/21R277). Written informed consent was obtained from all participants prior to inclusion in the study. Experiments using NOD/Shi-scid-IL2rγ^{null}-hIL-4 Tg (NOG; formal name, NOD.Cg-Prkdc^{scid}Il2rg^{tm1Sug}/ShiJic) (NOG-hIL4-Tg) mice were approved in compliance with the Guidelines for the Care and Use of Laboratory animals, and all animal studies were approved by the committees of the Tokai University School of Medicine. The numbers of approval are #22-011-27R1, #21R277, #235020. NOG-hIL4-Tg mice were maintained at Tokai University School of Medicine under specific pathogen-free conditions. DNA was extracted from ear tissues collected at the time of genotyping. Offspring expressing the human IL-4 transgene were identified as previously described.[4] The plasma concentration of human IL-4 (hIL-4) was measured as described below. During the experiments, all mice were housed under specific pathogen-free conditions in an animal facility at the CLEA or Tokai University School of Medicine. Freshly prepared PBMCs were used for experiments. Peripheral blood samples (approximately 30 mL) were collected from 4 healthy human donors without a history of malignancy into Vacutainer ACD tubes containing heparin (Becton Dickinson, NJ, USA). The samples were immediately placed in 10 mL of Ficoll-Paque PLUS (Cytiva, London, UK). The PBMCs were isolated by density centrifugation (800 ×*g* for 30 min at 20°C) and washed with phosphate-buffered saline (PBS) (475 ×*g* for 5 min at 4°C). The PBMCs (5×10^6 cells) were intravenously injected into 8-week-old NOG and NOG-hIL-4-Tg mice.

6-2. Immunization

All vaccines were administered within one week after preparation, during which stability was confirmed. Each group of four NOG-hIL-4-Tg (9 weeks of age) was inoculated intraperitoneally (i.p.). Because the vaccines were liquid type and not emulsified, they had a risk of leaking during subcutaneous administration. Given that both intraperitoneal and subcutaneous administration elicit similar levels of specific antibody production in wildtype mice,[5] we employed intraperitoneal administration to precisely administer the designated quantity to each mouse. The immunization schedule included boosting each mouse three times on Days 0, 14, and 28, respectively. Blood was collected with heparin from each mouse on Day 35, and was centrifuged to obtain plasma. Samples collected on Day 35 were stored at -80 °C for subsequent analyses.

6-3. ELISA

<Protocol>

Immuno Breakable Modules Clear, C8 LockWell, MediSorp (446470, Thermo Fisher Scientific, Massachusetts, US) were coated with a solution of the CH401-Multiple Antigenic Peptide (CH401-MAP, 2 µg /mL, 50 µL per well) in the coating buffer (50 mM carbonate, pH 9.5) at 4 ºC for 16 h. Nonspecific sites were blocked with 1% (w/v) BSA (9018A3733-50G, Sigma Aldrich, St. Louis, US) in PBS at pH 7.4 at 37 ºC for 2 h, then washed three times with PBS using ImmunoWash™ 1575 microplate washer (170-7009JA, Bio-Rad Laboratories, California, US). Subsequently, an individual mouse plasma with dilutions at 1:125 in PBS containing 1% BSA

was added to the coated plates (50 µL per well). The plates were incubated at 25 ºC for 2 h and then washed four times with PBS containing 0.05 % Tween-20 (PBST) using a plate washer. It was incubated at room temperature for 1 h with 50 µL of a 1:1000 diluted solution of biotin-conjugated goat anti-human IgG (H+L) (A80-219B, Bethyl Laboratories, Inc., Texas, US), goat anti-human IgM (A80-200B, Bethyl Laboratories, Inc.) antibody. After the plates were washed five times with PBST using a plate washer, 50 µL of 1:1000 diluted HRP-avidin (405103, BioLegend, California, US) was added and incubated at room temperature for 1 h in the dark. After the plates were washed seven times with PBST using a plate washer, 100 µL/well of 3,3',5,5'-tetramethylbenzidine (TMB) solution was added and incubated for 30 min at room temperature in the dark. 2 M aqueous sulfuric acid (50 µL per well) was subsequently added to stop the colorimetric reaction. Optical density (OD) was measured at 450 nm on a plate reader (Versa MAX, Molecular Devices, California, US).

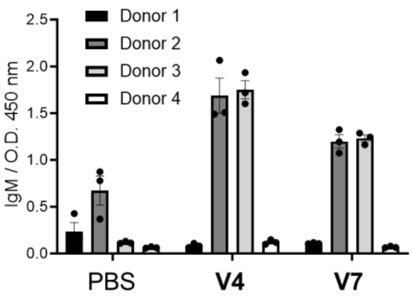

Figure S43. Anti-CH401 IgM titers of PBS/**V4**/**V7**-vaccinated NOG-hIL4-Tg mice (donor 1-4). Each dot represented the result from three experiments. The bar chart represented the average of the dots. The error bar represented the standard deviation of the mean.

6-4. Splenocyte analysis

<Protocol>

Splenocytes were collected and prepared as descrived in section 4. They were washed with PBS containing 0.05% (w/v) sodium azide twice. 5×10^5 spleen cells in PBS (50 µL) were stained with the fluorochrome-conjugated anti-human monoclonal antibodies in PBS containing 1% (w/v) BSA and 0.05% (w/v) sodium azide. After incubation for 20 min on ice in the dark, the stained spleen cells were washed with 1mL of PBS buffer and suspended in PBS containing 0.5% (w/v) BSA and 50 mM EDTA (pH 7.0). The stained cells were analyzed using BD FACS Fortessa ™ (BD Biosciences). The analysis of cell phenotypes was performed using FlowJo v10 software.

<B cell analysis>

Target/dye	Isotype	Company	Clone	Cat #	Dilution
CD5/PE-Cy7	Mouse IgG1, κ	BioLegend	UCHT2	300622	1:200
CD19/APC-Cy7	Mouse IgG1, κ	BioLegend	HIB19	302218	1:100
CD27/PE	Mouse IgG1, κ	BD Biosciences	M-T271	555441	1:200
CD38/Alexa Fluor 700 (AF700)	Mouse IgG1, κ	BioLegend	HIT2	303524	1:100
CD45/Pacific blue (PB)	Mouse IgG1, κ	BD Biosciences	HI30	304022	1:500

<CD4$^+$/CD8$^+$ T cell analysis>

Target/dye	Isotype	Company	Clone	Cat #	Dilution
CD3/FITC	Mouse IgG1, κ	BioLegend	UCHT1	300406	1:100
CD4/APC	Mouse IgG1, κ	BioLegend	RPA-T4	300514	1:500
CD8/Alexa Fluor 700 (AF700)	Mouse IgG1, κ	BioLegend	HIT8a	300920	1:50
CD19/APC-Cy7	Mouse IgG1, κ	BioLegend	HIB19	302218	1:100
CD25/PE	Mouse IgG1, κ	BioLegend	BC96	302606	1:100
CD45/Pacific blue (PB)	Mouse IgG1, κ	BioLegend	HI30	304022	1:500
CD56/PE-Cy7	Mouse IgG2b, κ	BD Biosciences	NCAM16.2	335791	1:500
PD-1/PerCP-Cy5.5	Mouse IgG1, κ	BioLegend	EH12.2H7	329914	1:50

<Naïve T/Tcm/Tem/Te cell analysis>

Target/dye	Isotype	Company	Clone	Cat #	Dilution
CD3/FITC	Mouse IgG1, κ	BioLegend	UCHT1	300406	1:100
CD4/Brilliant violet 510 (BV510)	Mouse IgG2b, κ	BioLegend	OKT4	317444	1:50
CD8/Brilliant Ultra Violet 395 (BUV395)	Mouse IgG1, κ	RPA-T8	563795	563795	1:200
CD45/ Brilliant Ultra Violet 737 (BUV737)	Mouse IgG1, κ	BD Biosciences	HI30	748719	1:200
CD45RA//Brilliant Violet 650 (BV650)	Mouse IgG2b, κ	BioLegend	HI100	304136	1:200
CD62L/PE	Mouse IgG1, κ	BioLegend	DREG-56	304806	1:500

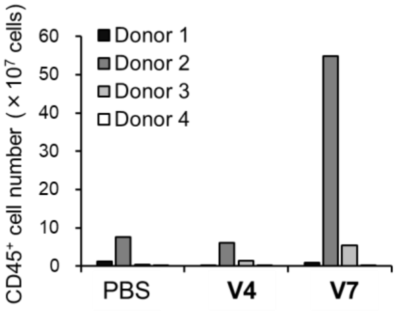

Figure S44. CD45$^+$ cell analysis in splenocytes of PBS/**V4**/**V7**-vaccinated NOG-hIL4-Tg mice (donor 1-4). CD45: human cell marker.

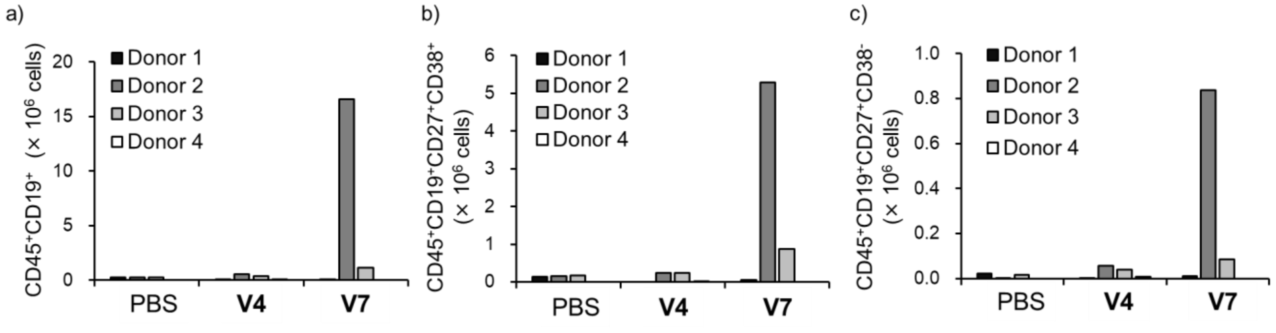

Figure S45. B cell analysis in splenocytes of PBS/**V4**/**V7**-vaccinated NOG-hIL4-Tg mice (donor 1-4). a) CD45$^+$CD19$^+$ B cell, b) CD45$^+$CD19$^+$ CD27$^+$ CD38$^+$ plasmablast B cell, c) CD45$^+$CD19$^+$ CD27$^+$CD38$^-$ memory B cell.

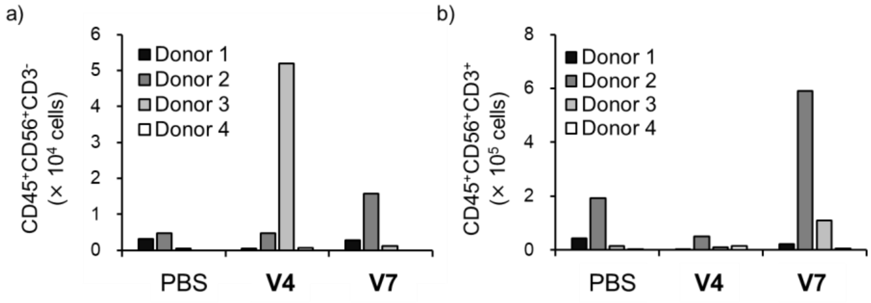

Figure S46. NK cell and NKT cell analysis in splenocytes of PBS/**V4**/**V7**-vaccinated NOG-hIL4-Tg mice (donor 1-4). a) CD45$^+$CD56$^+$ CD3$^-$ NK cell, b) CD45$^+$CD56$^+$ CD3$^+$ NKT cell.

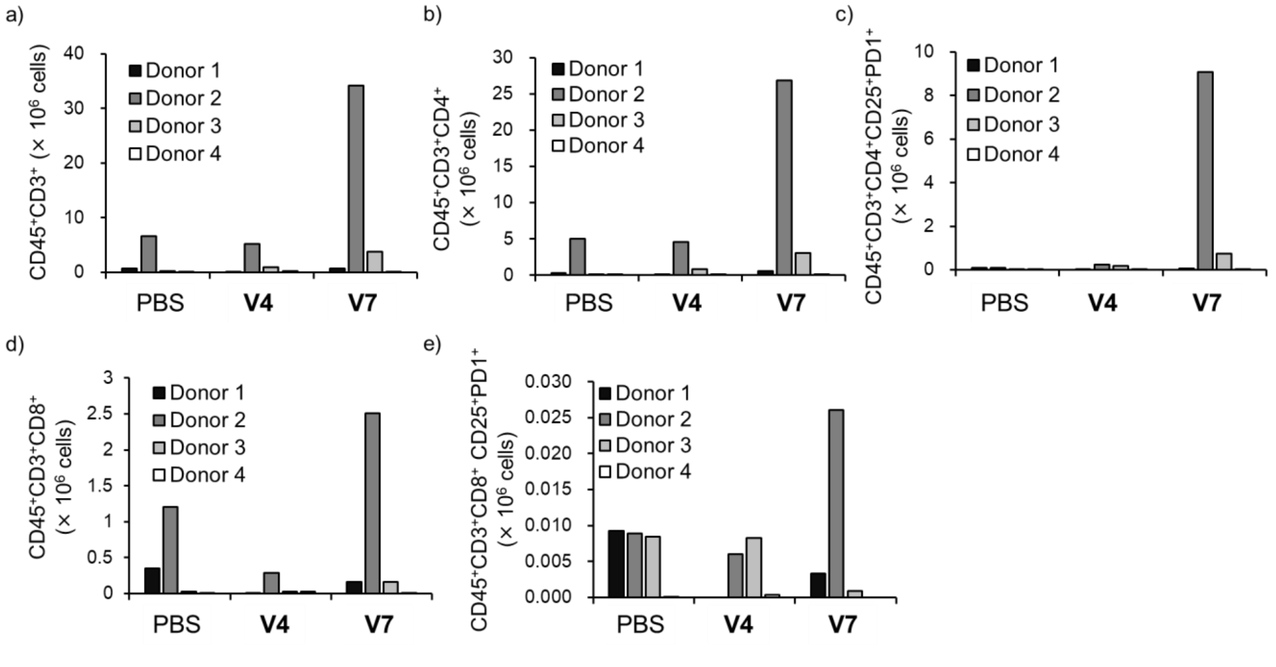

Figure S47. CD4$^+$/CD8$^+$ T cell analysis in splenocytes of PBS/**V4**/**V7**-vaccinated NOG-hIL4-Tg mice (donor 1-4). a) CD45$^+$CD3$^+$ T cell, b) CD45$^+$CD3$^+$CD4$^+$ Th cell, c) CD45$^+$CD3$^+$CD4$^+$CD25$^+$PD1$^+$ activated Th cell, d) CD45$^+$CD3$^+$CD8$^+$ killer T cell, e) D45$^+$CD3$^+$CD8$^+$CD25$^+$PD1$^+$ activated killer T cell.

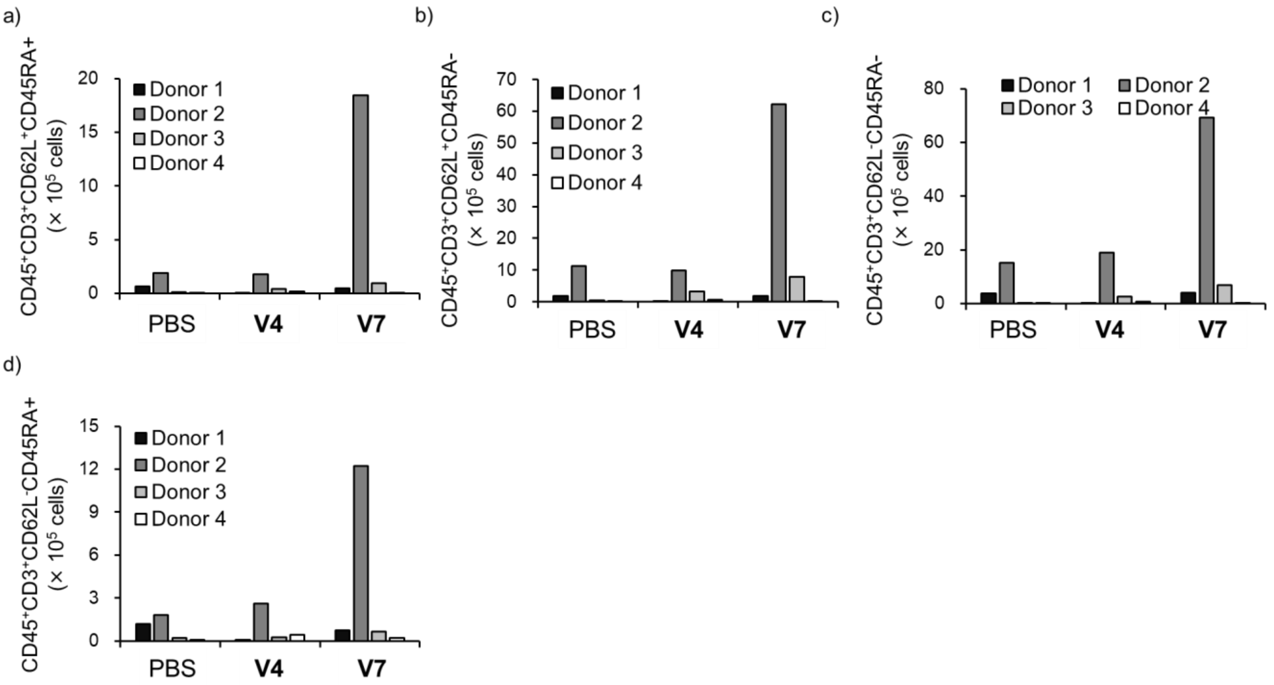

Figure S48. Naïve T/Tcm/Tem/Te cell analysis in splenocytes of PBS/**V4**/**V7**-vaccinated NOG-hIL4-Tg mice (donor 1-4). a) $CD45^+CD3^+CD62L^+CD45RA^+$ Naïve T cell, b) $CD45^+CD3^+CD62L^+CD45RA^-$ central memory T (Tcm) cell, c) $CD45^+CD3^+CD62L^-CD45RA^-$ effector memory T (Tem) cell, d) $CD45^+CD3^+CD62L^-CD45RA^+$ effector T (Te) cell.

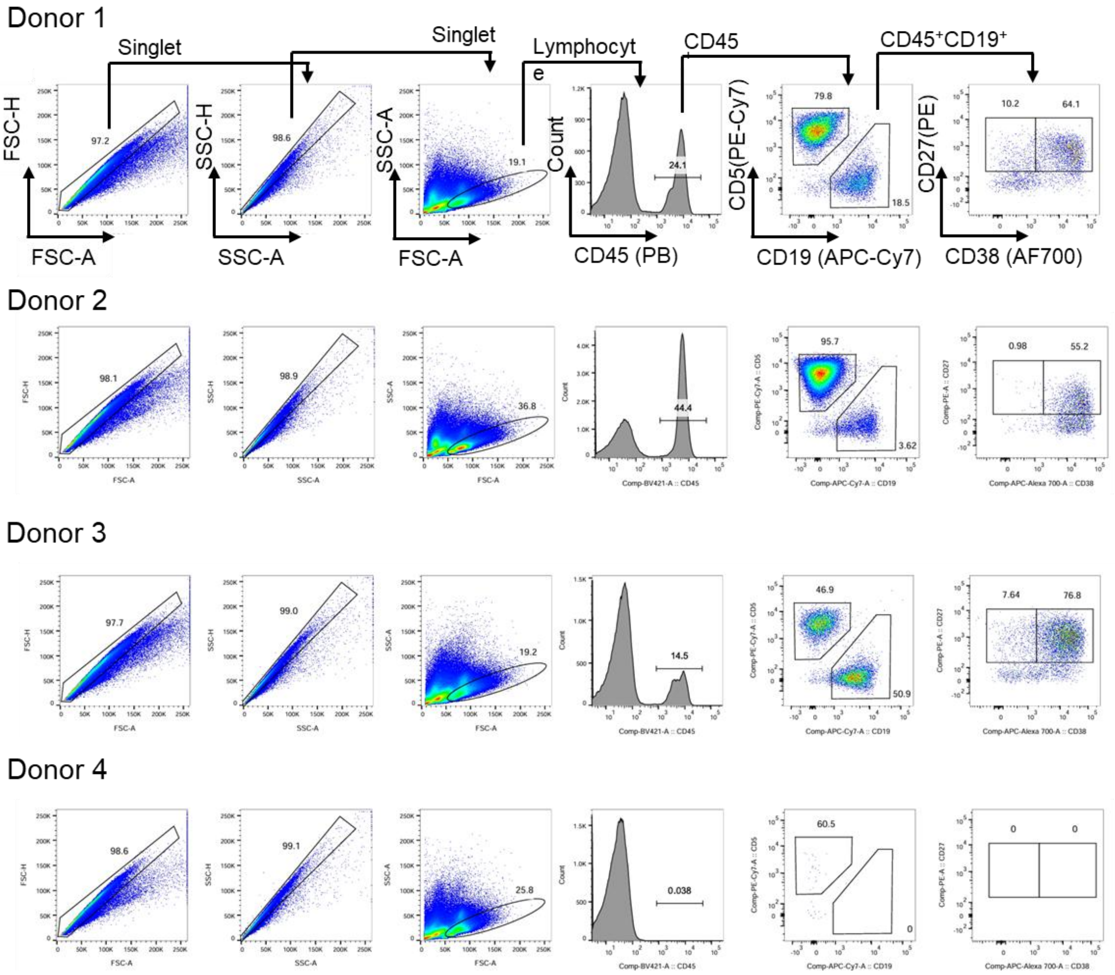

Figure S49. Gate for B cell analysis of splenocytes collected from mice NOG-hIL4-Tg immunized with PBS (n = 4).

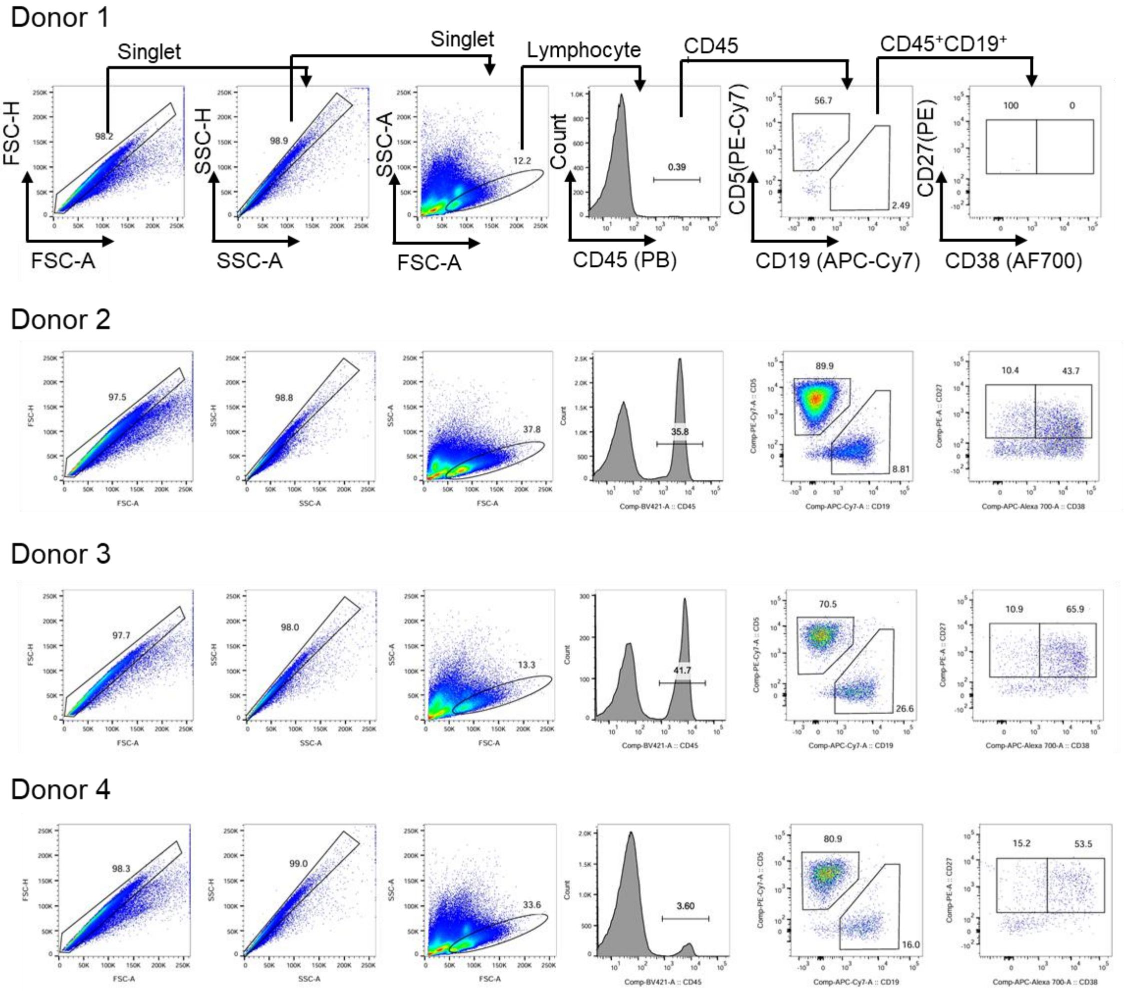

Figure S50. Gate for B cell analysis of splenocytes collected from mice NOG-hIL4-Tg immunized with **V4** (*n* = 4).

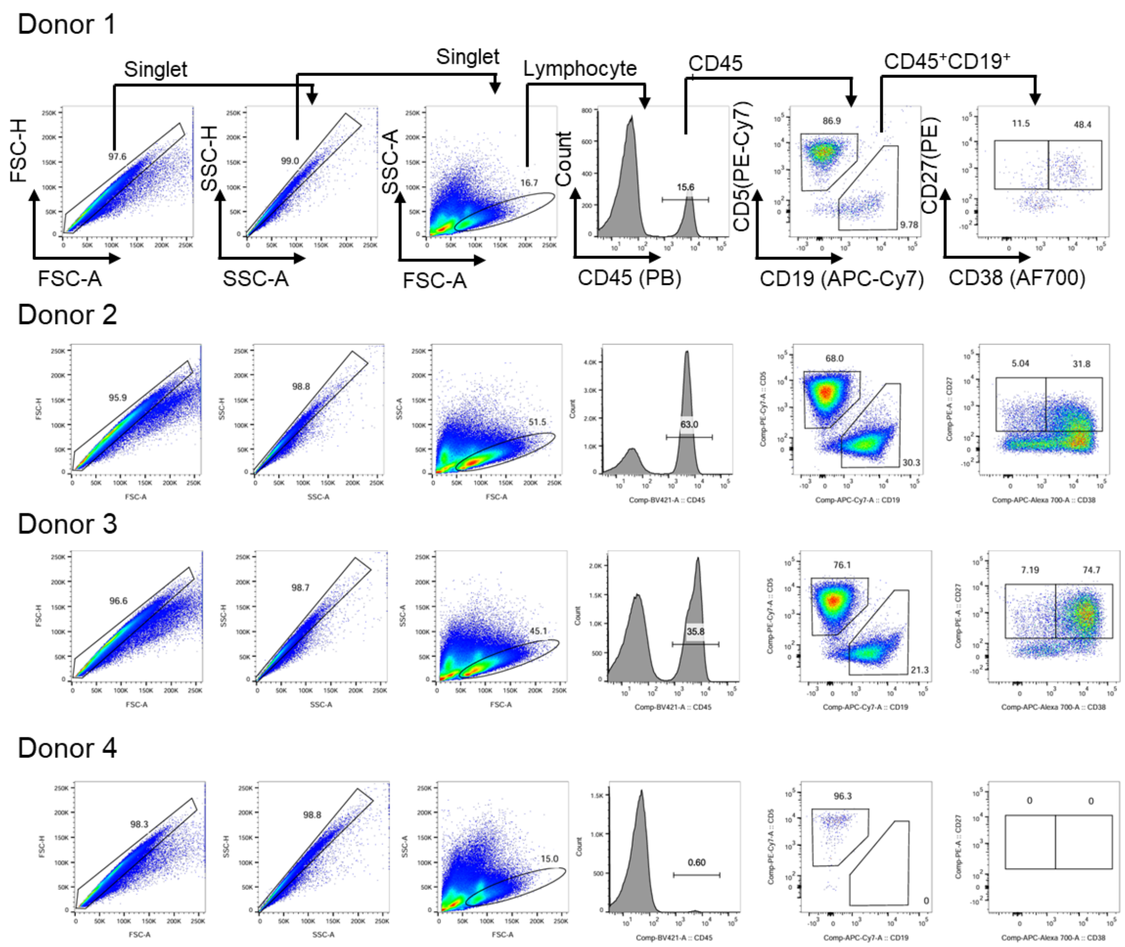

Figure S51. Gate for B cell analysis of splenocytes collected from mice NOG-hIL4-Tg immunized with **V7** (*n* = 4).

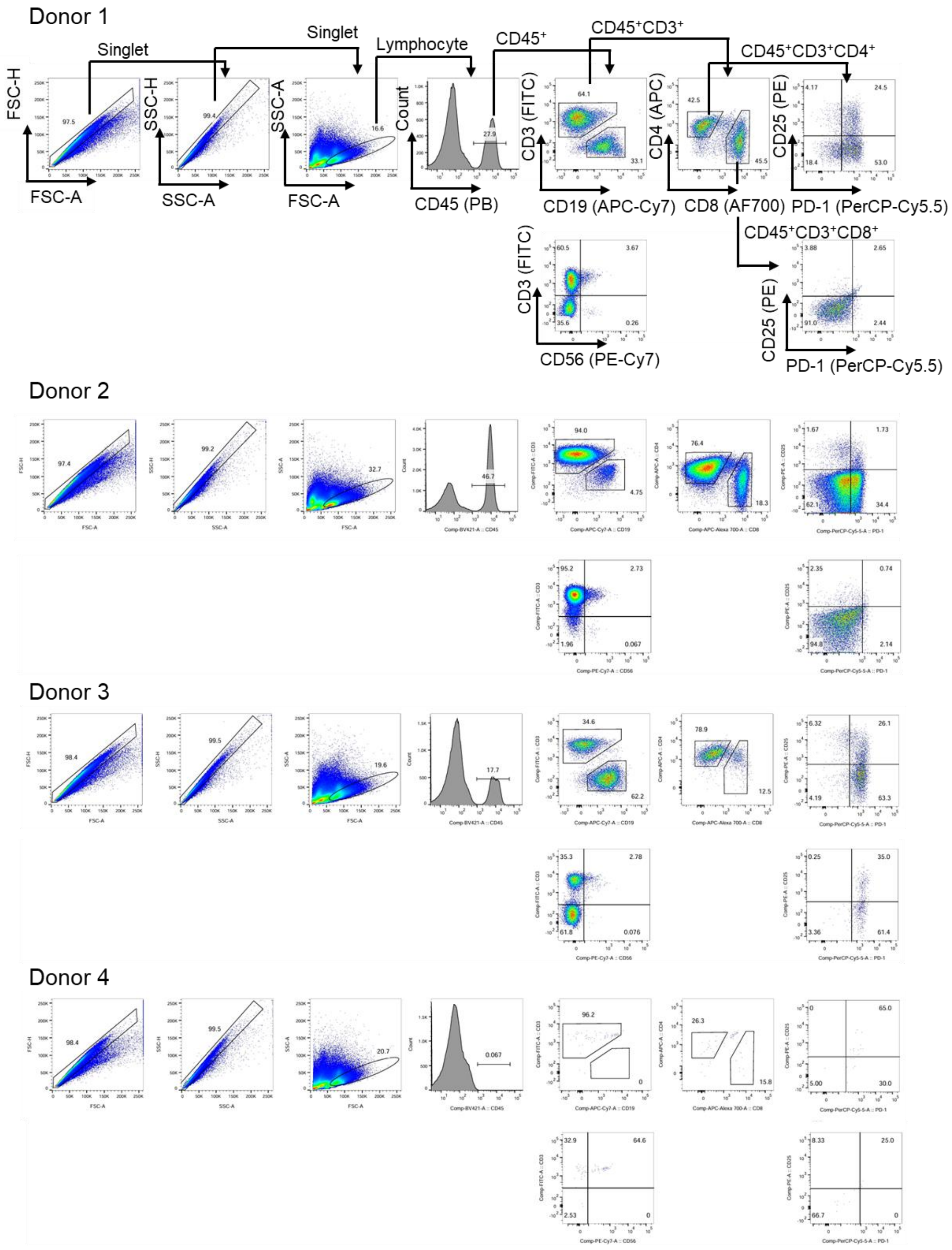

Figure S52. Gate for CD4^{+}/CD8^{+} T cell analysis of splenocytes collected from mice NOG-hIL4-Tg immunized with PBS (n = 4).

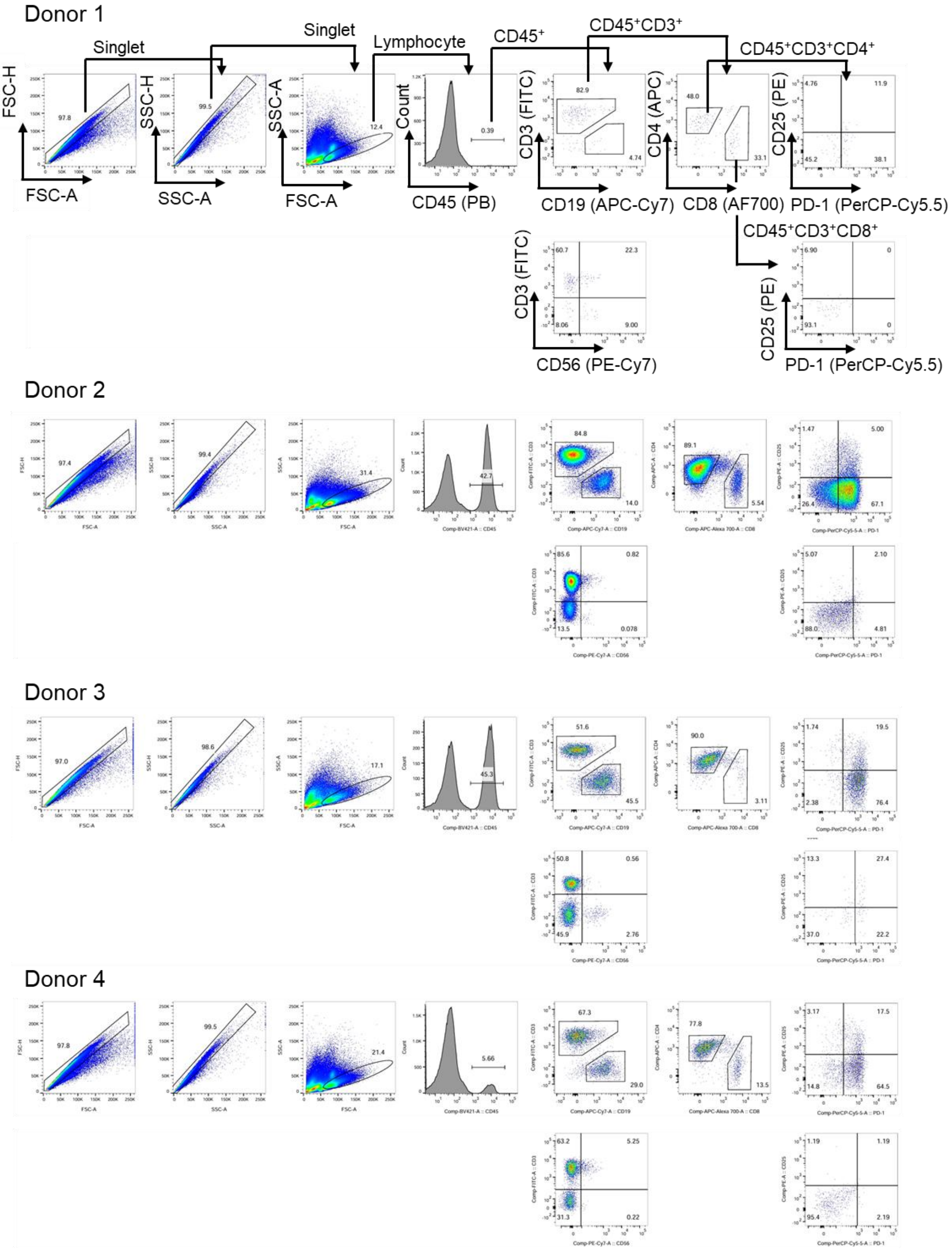

Figure S53. Gate for CD4$^+$/CD8$^+$ T cell analysis of splenocytes collected from mice NOG-hIL4-Tg immunized with **V4** (n = 4).

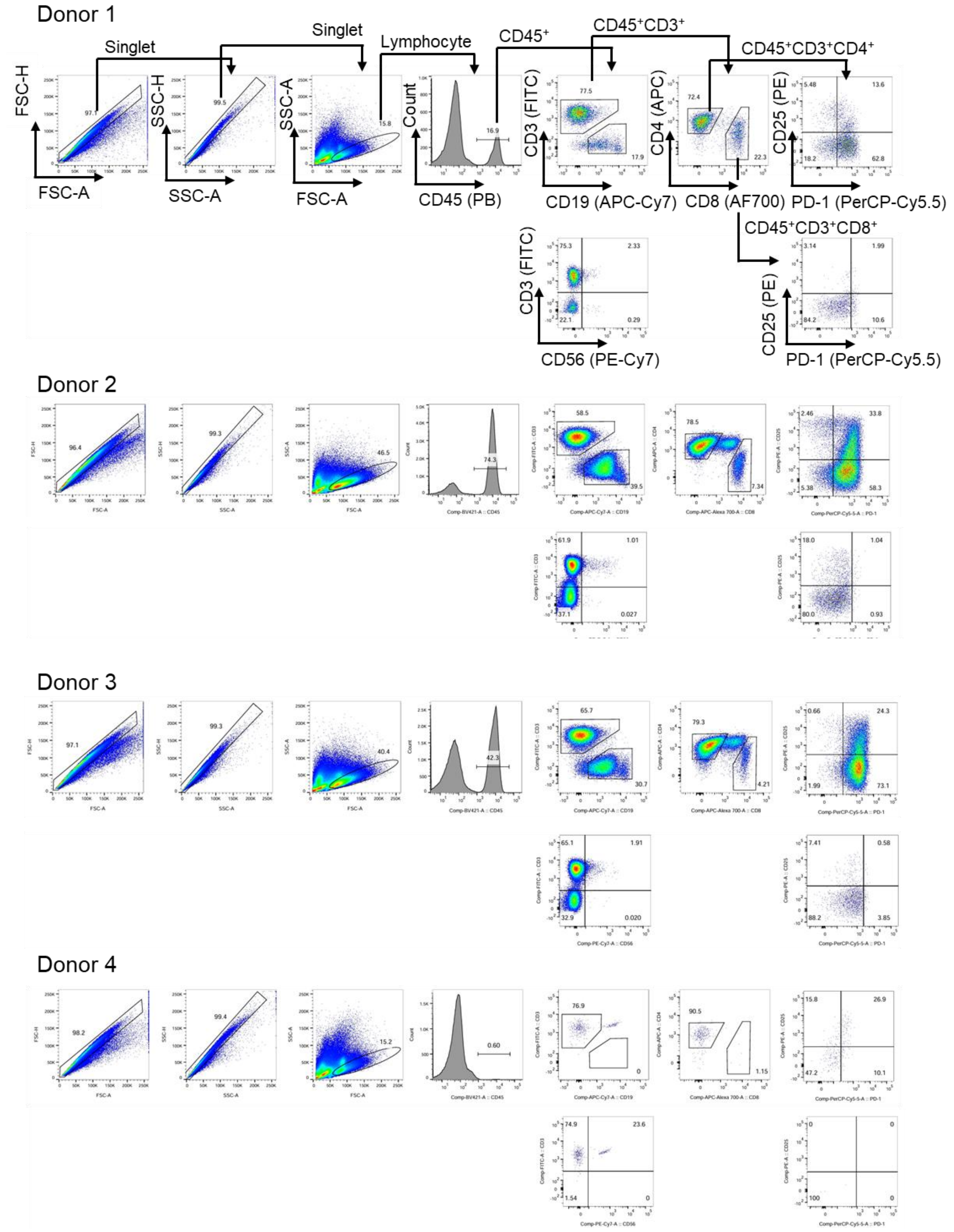

Figure S54. Gate for CD4^{+}/CD8^{+} T cell analysis of splenocytes collected from mice NOG-hIL4-Tg immunized with **V7** (n = 4).

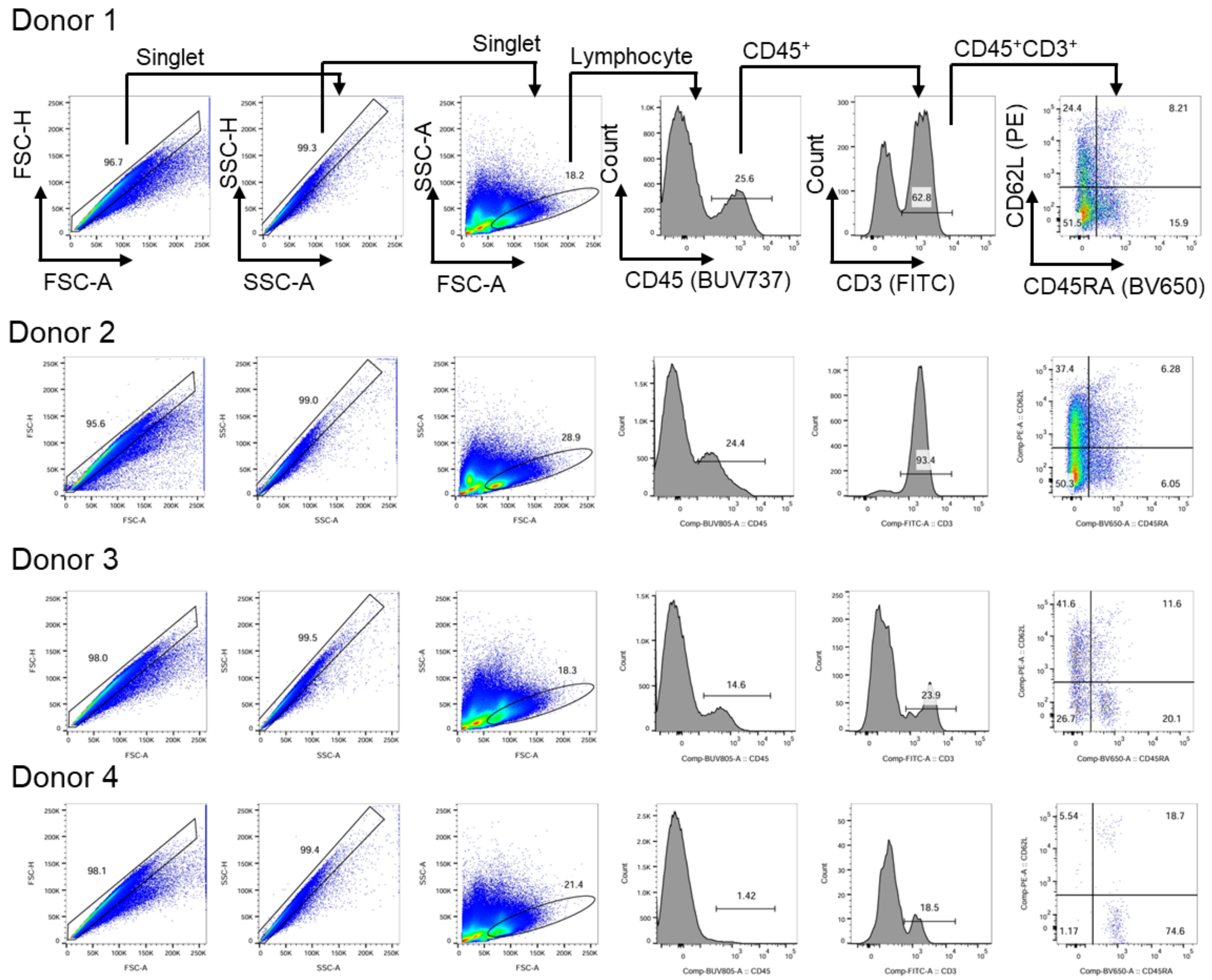

Figure S55. Gate for Naïve T/Tcm/Tem/Te cell analysis of splenocytes collected from mice NOG-hIL4-Tg immunized with PBS (n = 4).

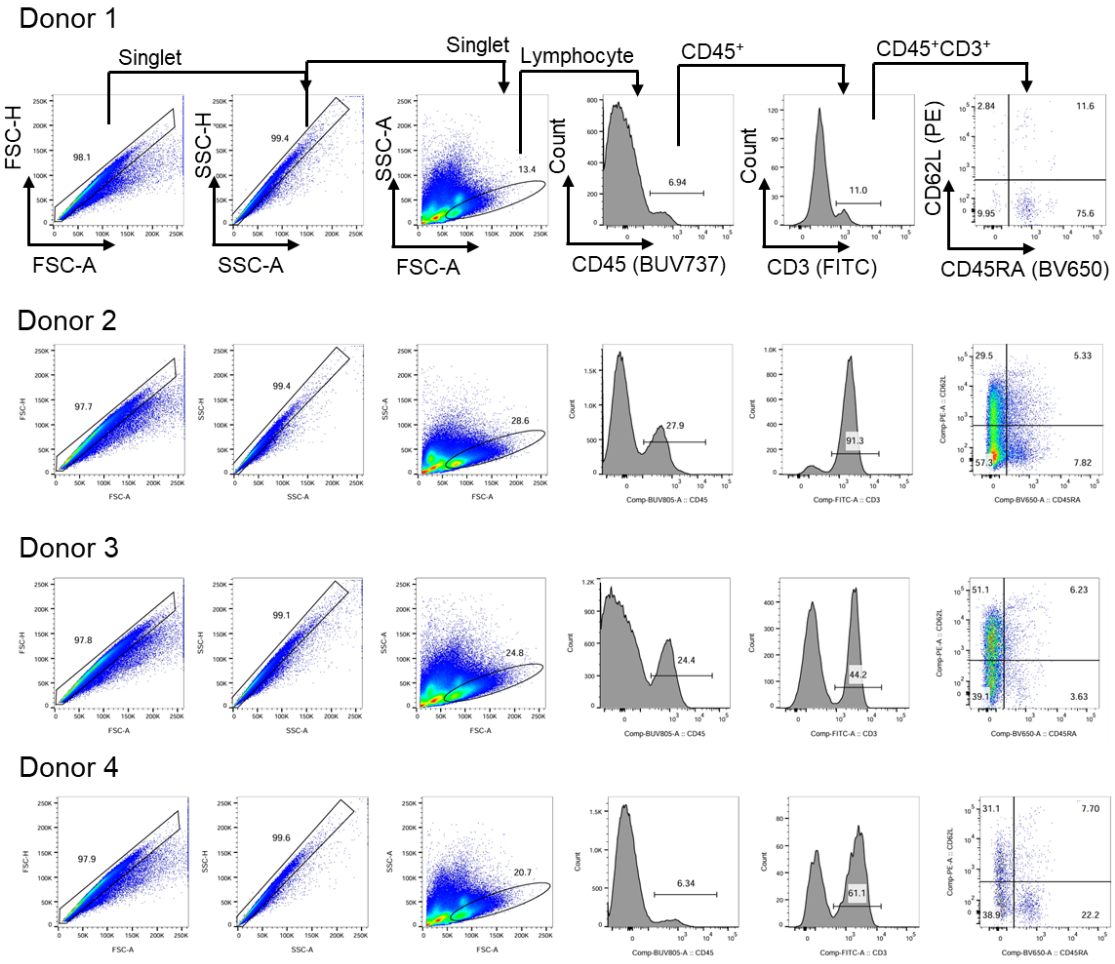

Figure S56. Gate for Naïve T/Tcm/Tem/Te cell analysis of splenocyte collected from mice NOG-hIL4-Tg immunized with **V4** (n = 4).

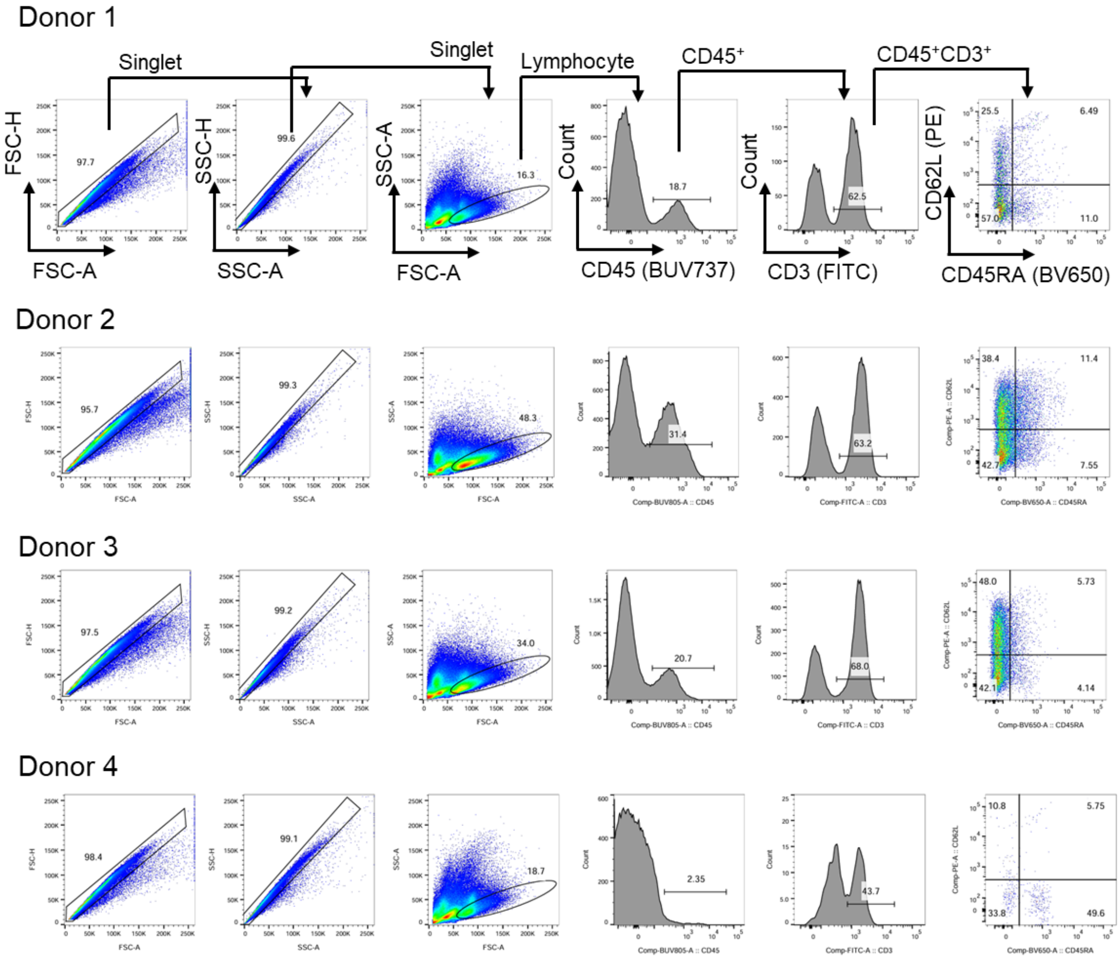

Figure S57. Gate for Naïve T/Tcm/Tem/Te cell analysis of splenocyte collected from mice NOG-hIL4-Tg immunized with **V7** (n = 4).

6-5. ELISpot

<Protocol>

ELISpot assays were performed using Immunospot® human IFN-γ/Granzyme B Double-color ELISPOT (Cellular Technology Ltd., Ohio, US) according to the manufacturer's instructions. 3×10^5 cells of each splenocyte collected from immunized NOG-hIL4-Tg mice were cultured in the absence/presence of anti-CD3 antibody/TSST-1/CH401 multiple antigenic peptide (CH401-MAP) in CTL-Test™ medium (200 µL per well, final concentration of CH401-MAP: 10 µg/mL) to secrete cytokines. Spots were scanned and counted using an ImmunoSpot® S6 analyzer (Cellular Technology Ltd.).

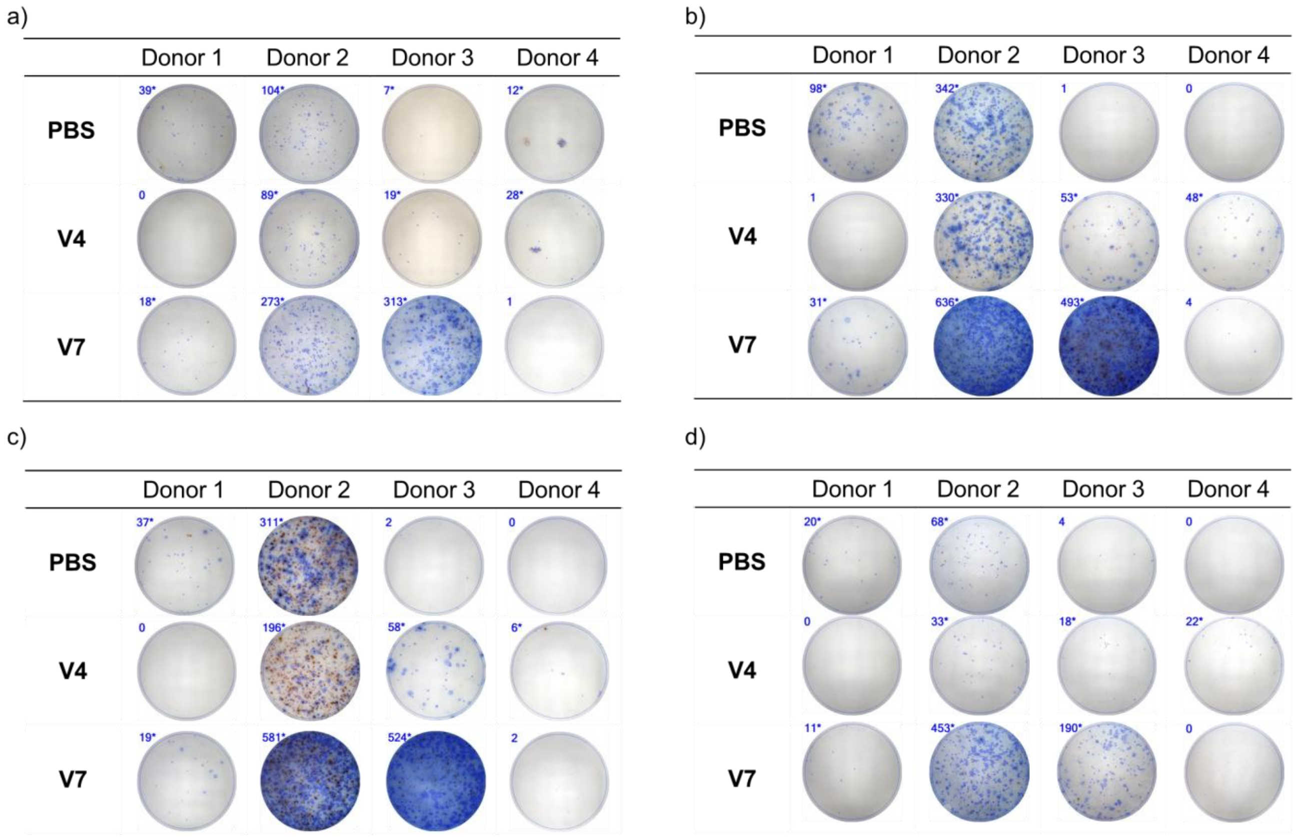

Figure S58. ELISpot results of Granzyme B (blue spot) from splenocytes harvested from PBS/**V4**/**V7**-vaccinated NOG-hIL4-Tg mice (donor 1-4). a) No stimulation, b) anti-CD3 antibody stimulation, c) TSST-1 stimulation, d) CH401-MAP stimulation. The number of spots was represented in the upper left of each well.

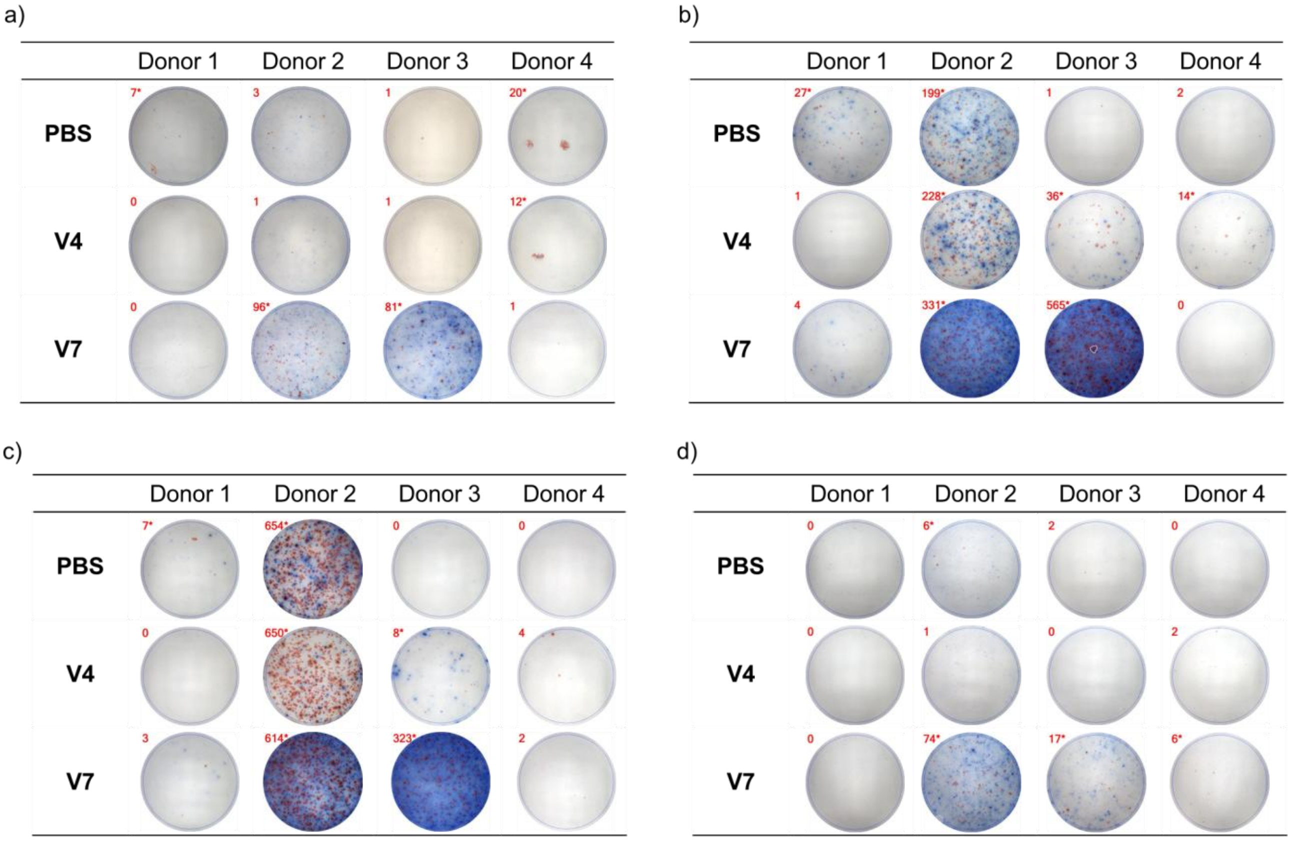

Figure S59. ELISpot results of IFN-γ (red spot) from splenocytes harvested from PBS/**V4**/**V7**-vaccinated NOG-hIL4-Tg mice (donor 1-4). a) No stimulation, b) anti-CD3 antibody stimulation, c) TSST-1 stimulation, d) CH401-MAP stimulation. The number of spots was represented in the upper left of each well.

6-6. Cytokine analysis using LEGENDplex

<Protocol>

5×10^5 cells of each splenocyte were cultured with 1µg/mL toxic shock syndrome toxin-1 (TSST-1, Toxin Technology Inc., Florida, USA) in RPMI-1640 containing 10% (v/v) FBS (120 µL per well) on IWAKI 24 well microplates (3820-024, AGC TECHNO GLASS Co., Ltd., Shizuoka, Japan). After incubation for 72 h at 37 °C and 5% CO_2, the supernatants were collected, and the cytokines in the supernatants were evaluated by LEGENDplex™ HU Th cytokine Panel (12-plex) w/FPV02 (741027, BioLegend, California, US) according to the manufacturer's instructions. The panel included IFN-γ, TNF-α, IL-2, IL-4, IL-5, IL-6, IL-9, IL-10, IL-13, IL-17A, IL-17F, IL-22. The data were acquired using BD FACSLyric™ (BD Biosciences). The acquired data were analyzed using LEGENDplex™ software (BioLegend).

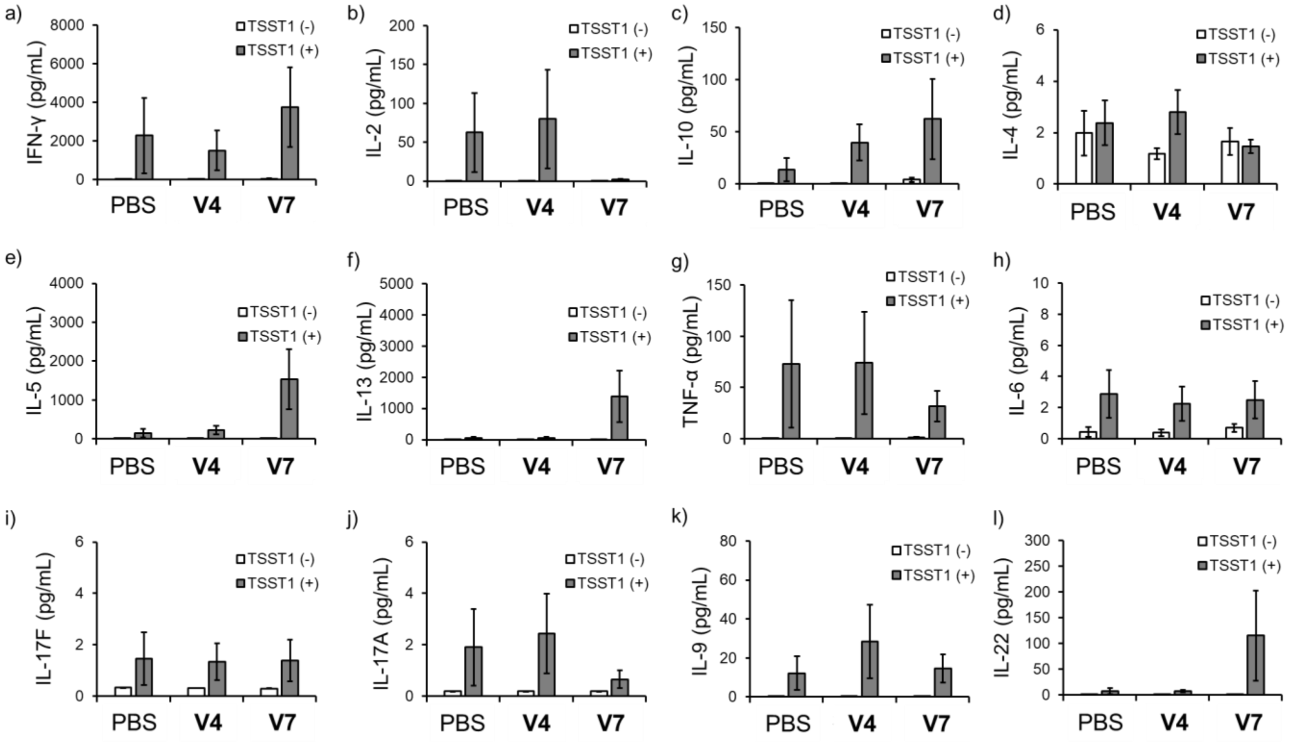

Figure S60. Cytokine profiles secreted by splenocytes collected from PBS/**V4**/**V7**-vaccinated NOG-hIL4-Tg mice (donor 1-4). a) IFN-γ, b) IL-2, c) IL-10, d) IL-4, e) IL-5, f) IL-13, g) TNF-α, h) IL-6, i) IL-17A, IL-17F, j) IL-9, k) IL-22. The data represent the results from 4 donors (n = 4). The bar chart represented the average of the results from 4 donors (n = 4). The error bar represented the standard deviation of the mean.

7. References

[1] T. Aiga, Y. Manabe, K. Ito, T.-C. Chang, K. Kabayama, S. Ohshima, Y. Kametani, A. Miura, H. Furukawa, H. Inaba, K. Matsuura, K. Fukase, *Angew. Chem. Int. Ed.* **2020**, *59*, 17705-17711.

[2] N. Kimura, M. Maeki, Y. Sato, Y. Note, A. Ishida, H. Tani, H. Harashima, M. Tokeshi, *ACS Omega* **2018**, *3*, 5044-5051.

[3] C. T. Inglut, A. J. Sorrin, T. Kuruppu, S. Vig, J. Cicalo, H. Ahmad, H.-C. Huang, *Nanomaterials* **2020**, *10*, 190.

[4] Y. Kametani, I. Katano, A. Miyamoto, Y. Kikuchi, R. Ito, Y. Muguruma, B. Tsuda, S. Habu, Y. Tokuda, K. Ando, M. Ito, *PLOS ONE* **2017**, *12*, e0179239.

[5] M. Leenaars, C. F. Hendriksen, *Altex* **1998**, *15*, 87.
